# Determinants of GPI-PLC Localisation to the Flagellum and Access to GPI-Anchored Substrates in Trypanosomes

**DOI:** 10.1371/journal.ppat.1003566

**Published:** 2013-08-22

**Authors:** Jack Sunter, Helena Webb, Mark Carrington

**Affiliations:** Department of Biochemistry, University of Cambridge, Cambridge, United Kingdom; Washington University School of Medicine, United States of America

## Abstract

In *Trypanosoma brucei*, glycosylphosphatidylinositol phospholipase C (GPI-PLC) is a virulence factor that releases variant surface glycoprotein (VSG) from dying cells. In live cells, GPI-PLC is localised to the plasma membrane where it is concentrated on the flagellar membrane, so activity or access must be tightly regulated as very little VSG is shed. Little is known about regulation except that acylation within a short internal motif containing three cysteines is necessary for GPI-PLC to access VSG in dying cells. Here, GPI-PLC mutants have been analysed both for subcellular localisation and for the ability to release VSG from dying cells. Two sequence determinants necessary for concentration on the flagellar membrane were identified. First, all three cysteines are required for full concentration on the flagellar membrane. Mutants with two cysteines localise predominantly to the plasma membrane but lose some of their flagellar concentration, while mutants with one cysteine are mainly localised to membranes between the nucleus and flagellar pocket. Second, a proline residue close to the C-terminus, and distant from the acylated cysteines, is necessary for concentration on the flagellar membrane. The localisation of GPI-PLC to the plasma but not flagellar membrane is necessary for access to the VSG in dying cells. Cellular structures necessary for concentration on the flagellar membrane were identified by depletion of components. Disruption of the flagellar pocket collar caused loss of concentration whereas detachment of the flagellum from the cell body after disruption of the flagellar attachment zone did not. Thus, targeting to the flagellar membrane requires: a titratable level of acylation, a motif including a proline, and a functional flagellar pocket. These results provide an insight into how the segregation of flagellar membrane proteins from those present in the flagellar pocket and cell body membranes is achieved.

## Author Summary

African trypanosomes are unicellular parasites with a single flagellum that maintain a persistent infection through antigenic variation based on changes in a densely packed cell surface coat of variant surface glycoprotein (VSG). The cells also contain an enzyme, GPI-PLC, able to shed the VSG from the cell surface. However, the activity is regulated and substantial shedding only occurs from dying cells. The GPI-PLC is found predominantly on the membrane of this flagellum. Here, we have investigated the relationship between this subcellular localisation and VSG shedding ability of the GPI-PLC. We found that two motifs are important: a cluster of three cysteines that are modified by the addition of fatty acids and a proline, mutation of which caused the redistribution of GPI-PLC from the flagellar to the plasma membrane. Localisation of GPI-PLC to the plasma membrane is necessary for GPI-PLC to access the VSG in dying cells. Finally, the correct localisation of the GPI-PLC was dependent on a functional flagellar pocket. These results have provided a significant and exploitable insight into the regulation of GPI-PLC and more generally into how proteins are targeted to the flagellum membrane.

## Introduction

The external cell surface of the mammalian bloodstream form of African trypanosomes is covered with a densely packed protein coat predominantly composed of a single polypeptide species, the variant surface glycoprotein (VSG) [1], [2]. The VSG is central to the interaction with the host: it functions in both a population survival strategy through antigenic variation and an individual cell survival strategy though rapid endocytosis, removal of bound antibody, and recycling back to the cell surface [3], [4], [5]. The VSG coat is essential and loss of VSG synthesis causes a growth arrest in culture and cell death in an animal model [6]. The VSG is attached to the external face of the plasma membrane through a C-terminal glycosylphosphatidylinositol (GPI) anchor [7]. Bloodstream form trypanosomes contain a GPI-phospholipase C (GPI-PLC) [8], [9], [10] and when plasma membrane integrity is compromised, for example by hypotonic lysis, hydrolysis by GPI-PLC releases all VSG from the plasma membrane within five minutes [10], [11]. However, in a population of cells, the half-life of VSG is ∼30 hours, equivalent to several cell generations [12], [13], indicating that there is very tight regulation of GPI-PLC activity and/or access to VSG in live cells

The *GPI-PLC* gene is not essential but acts a virulence factor as a null (−/−) mutant was attenuated in mice [14]. The attenuation may be caused by the failure to release VSG from trypanosomes killed by the host immune response. Release of the VSG from dying trypanosomes into the host bloodstream may cause the VSG antibody response to be directed towards novel epitopes on the released VSG and away from expanding populations of cells expressing novel VSGs [15]. However, no definitive function for GPI-PLC has been identified.

GPI-PLC behaves as an integral membrane protein [9], [16]: it has neither an N-terminal signal peptide nor a transmembrane domain [17] but contains a short motif, 268 ACCGACP 274 (abbreviated to CCGAC), which contains three cysteine residues that were shown to be modified by palmitoylation when GPI-PLC was expressed in *Xenopus* oocytes [18]; furthermore, native GPI-PLC is acylated [19]. In trypanosomes, when acylation was prevented through expression of a GPI-PLC mutant transgene containing the sequence 268 ASRGARP 274 in a trypanosome *GPI-PLC* −/− background, there was no release of VSG on hypotonic lysis [18].

The subcellular localisation of GPI-PLC has been investigated and two overlapping but distinct results were obtained [20]. Immunofluorescence localised GPI-PLC to a linear array along the flagellum between the paraflagellar rod and flagellar attachment zone (FAZ) and evidence was presented for localisation to the external face of the plasma membrane. In contrast, GPI-PLC tagged at the C-terminus with eYFP localised to the plasma membrane, being more concentrated on the flagellar membrane than on the cell body [20]. In trypanosomes, the flagellar membrane is one of three discrete domains of the plasma membrane, the other two being the cell body and the flagellar pocket (FP) [21]. The protein complement in these domains is dominated by the VSG, but each domain also contains a set of unique proteins [20], [22], [23]. The three domains are demarcated by two structures: firstly by the flagellar pocket collar (FPC), a ring at the neck of the FP that marks the boundary between the cell body and FP membranes; and second by the collarette, which marks the boundary between the flagellar and FP membranes at the point at which the flagellum enters the FP [21]. The FP is the only site of exocytosis and endocytosis [24] and all components of the flagellar and cell body membranes added through vesicular transport pass through the FP. Subsequent sorting in the FP must ensure that components reach their correct destination. The FPC may act as a diffusion barrier to maintain the distinct membrane composition of the flagellum, for example the higher concentration of GPI-PLC [20]. One component of the FPC, BILBO1, has been characterised: knockdown results in the loss of both the FPC and the FP at the newly synthesised flagellum and subsequent cell death [25].

Acylation is a common but not universal theme in membrane proteins that localise to flagella or cilia [26], when acylation occurs it is necessary for localisation [27], [28], [29], [30]. However, in some proteins acylation is not sufficient and other amino acid motifs are also required for efficient targeting [28]. Here, the relationship between subcellular localisation and access to the VSG substrate has been investigated through expression of GPI-PLC mutants both to provide information about the regulation of GPI-PLC and also to identify determinants necessary for concentration on the flagellar membrane that may be applicable to a wider range of proteins. When all the cysteines within the CCGAC motif are mutated to serines, GPI-PLC is still enzymically active but is now cytoplasmic and unable to release the VSG coat on hypotonic lysis. The correct pattern of modifications of the CCGAC motif is required for successful trafficking of GPI-PLC to the flagellar membrane: as the number of cysteines is reduced, GPI-PLC becomes localised to the endomembranes between the nucleus and flagellar pocket. However, acylation of the cysteine residues is not sufficient for flagellar localisation: mutation of a single proline results in the failure to concentrate on the flagellar membrane. The concentration on the flagellar membrane requires a functional FPC but does not require flagellar attachment to the cell body.

## Results

### Comparison of activities of GPI-PLC and GPI-PLC-eYFP

Hydrolysis of GPI-anchors by GPI-PLC results in the release of diacylglycerol and the formation of inositol 1, 2 cyclic phosphate on the GPI-glycan. The cyclic phosphate is part of the cross-reacting determinant (CRD), an epitope formed by GPI-anchor hydrolysis by GPI-PLC [31], [32], [33]. In vitro, two assays are used to analyse GPI-PLC activity in trypanosomes. First, after detergent lysis, activity is determined by following the appearance of the CRD epitope on the VSG: this assay is independent of any regulation based on spatial segregation. In the second assay, hypotonic lysis is used to rupture the plasma membrane and hydrolysis of the GPI-anchor is detected by separating the reaction into pellet and supernatant fractions; before GPI-hydrolysis membrane-attached VSG is in the pellet fraction and afterward in the supernatant fraction. In this assay, only VSG present on the plasma membrane is hydrolysed; newly synthesised VSG en route to the plasma membrane is not a substrate, which is assumed to be due to lack of access as all VSG is hydrolysed on detergent lysis [34]. The hypotonic lysis assay measures not only the enzymic activity of GPI-PLC, but also its access to plasma membrane VSG. Electron micrographs of hypotonically lysed cells showed cell ghosts with clear holes in the plasma membrane [35], and the assay is used as the best approximation of immune system-mediated rupture. Using these assays the *GPI-PLC* −/− cell line is unable to hydrolyse the GPI-anchor [14].

**Figure 1:**
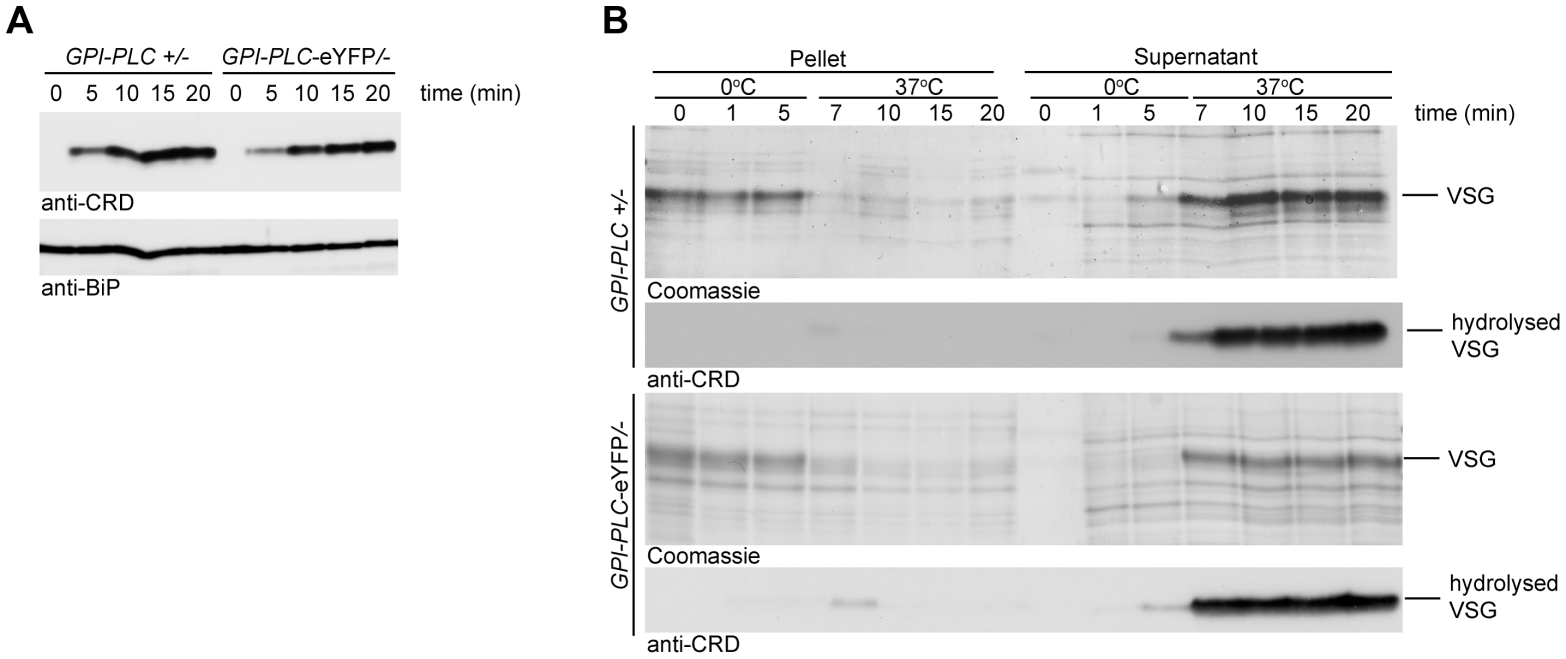
Wild type GPI-PLC and GPI-PLC-eYFP hydrolyse the VSG GPI-anchor at similar rates. (A) Cell lines modified to contain a single copy of the wild type *GPI-PLC* gene (*GPI-PLC*/−) or a single copy modified with a C-terminal eYFP tag (*GPI-PLC-eYFP*/−) were lysed with 0.5% triton X-100 and samples removed over a time course. The appearance of the CRD epitope in the lysates was used as a measure of the product of GPI-PLC action; anti-BiP was used as a loading control. 2×10^6^ cell equivalents were loaded in each track. (B) The same cell lines were hypotonically lysed on ice for 5 minutes and then warmed to 37°C. Samples were taken over this time course and centrifuged to pellet cell bodies. Pellet and supernatant fractions were then analysed by Coomassie stained gels and Western blots probed with anti-CRD. 1×10^6^ cell equivalents were loaded in each track.

To determine whether the C-terminal eYFP tag affected the activity of GPI-PLC, the relative rate of VSG GPI-anchor hydrolysis after detergent and hypotonic lysis was determined in two cell lines, *GPI-PLC*/− and *GPI-PLC-eYFP*/−. The cell lines were made by first deleting one allele [14] to make the *GPI-PLC*/− cells and then modifying the remaining allele to make *GPI-PLC-eYFP*/− cell lines [36]. Expression was examined by Western blotting using GPI-PLC antiserum: both proteins had the expected relative molecular mass and similar expression levels (Figure S1A). Both were expressed at lower levels than the parental GPI-PLC +/+ cell line [37], [38]. The rate of hydrolysis of the VSG GPI-anchor was similar in both detergent and hypotonic lysis assays of both cell lines (Figure 1), indicating that the eYFP tag did not affect the ability of GPI-PLC to access or hydrolyse its VSG substrate.

**Figure 2:**
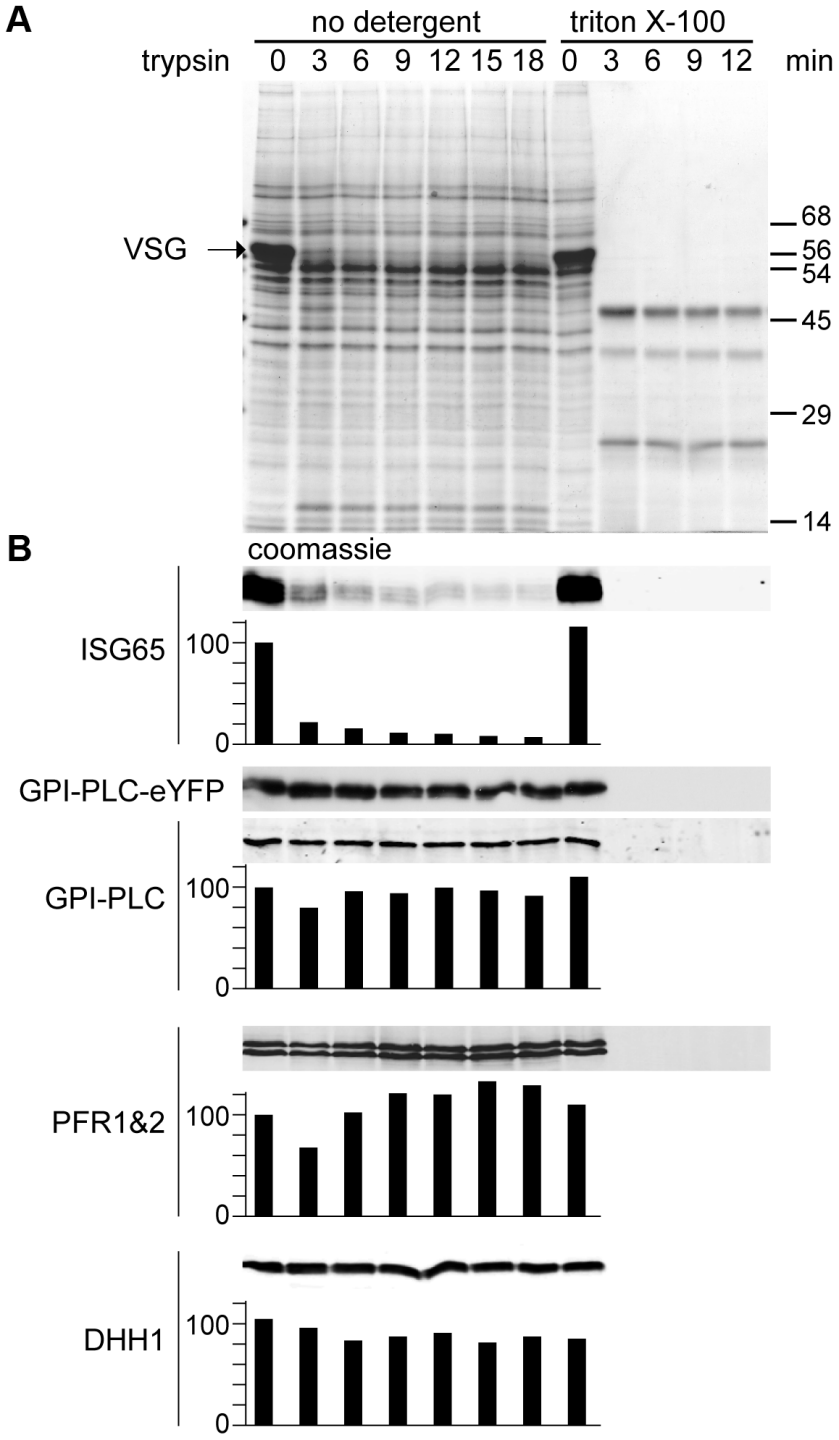
GPI-PLC is not sensitive to trypsin digest in live cells. A cell line modified to contain one copy of the wild type *GPI-PLC* gene and one copy modified with a C-terminal eYFP tag (GPI-PLC-eYFP/+) was treated with exogenous trypsin and samples were removed over a time course for analysis by SDS-PAGE and Western blotting. A parallel time course after 0.5% triton X-100 addition was used as a control for trypsin sensitivity. (A) Coomassie stained gel of a trypsin digest time course of *GPI-PLC*-eYFP/+ cells. Note the almost complete digestion of VSG by 3 minutes. (B) Western blots with relative quantitation below, setting the zero time point at 100. Quantitation used an Odyssey Infrared Imaging System and associated software. However, the expression levels of GPI-PLC-eYFP were too low for reliable detection using fluorescent secondary antibodies and were instead detected by chemiluminescence; this was not quantitated. ISG65 was mostly digested within 3 minutes. GPI-PLC-eYFP and GPI-PLC were stable over the time course but were digested within 3 minutes when detergent was included as were the flagellar proteins PFR1 and 2 and the cytoplasmic protein DHH1. 2×10^6^ cell equivalents were loaded in each track.

The membrane topology of GPI-PLC has been the subject of much discussion, with some evidence presented that some or all of the GPI-PLC is on the external face of the plasma membrane despite the absence of an obvious signal sequence [20]. A comparison of the membrane topology of tagged and untagged GPI-PLC was performed by determining the sensitivity to trypsin after addition to live cells expressing both wild type and eYFP tagged forms of the protein (Figure 2). The cell line for these experiments was made by modifying one of the *GPI-PLC* alleles with eYFP in wild type cells [36]. Expression was dependent on endogenous transcription and total GPI-PLC protein levels were similar to wild type. Two proteins present on the external face of the plasma membrane were used as controls: VSG and ISG65 [39]. The cytoplasmic controls were the paraflagellar rod proteins PFR1 and PFR2 [40] as well as the cytoplasmic RNA helicase DHH1 [41]. VSG and ISG65 were digested rapidly whereas the cytoplasmic proteins were stable until the endpoint at 18 minutes. Almost complete digestion of ISG65 was unexpected as a fraction resides in the endosomal membrane system [42]; it is possible that either recycling of ISG65 back to the cell surface continued during the trypsin digest and/or trypsin gained access to the endosomal compartment. Both forms of GPI-PLC behaved exactly the same as cytoplasmic proteins, suggesting that the majority of the protein is on the internal, not external, face of the plasma membrane. However, this assay would not have detected a small fraction of externally disposed GPI-PLC. All proteins were digested within three minutes if the plasma membrane was disrupted with detergent.

### Expression of GPI-PLC-eYFP cysteine motif mutants

GPI-PLC-eYFP was used as a reporter for mutational analysis of GPI-PLC.

**Figure 3:**
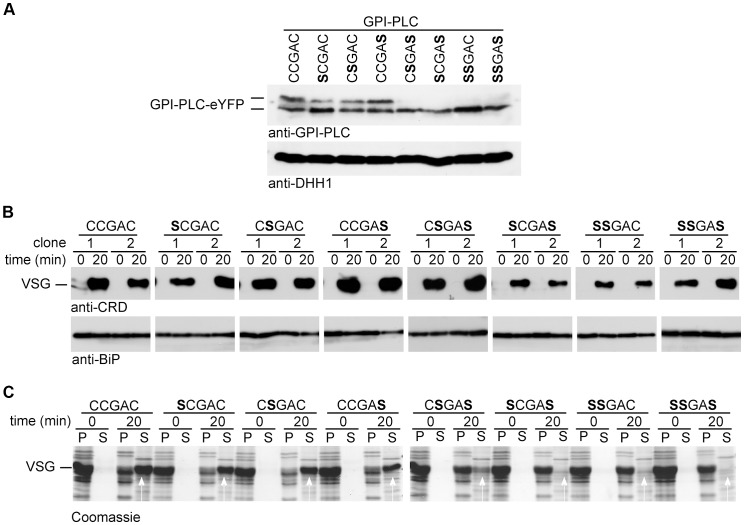
Cysteine mutants are enzymically active but unable to access VSG on hypotonic lysis. Cell lines modified to contain a single copy of the wild type (CCGAC) or cysteine mutant *GPI-PLC* all modified with a C-terminal eYFP tag were assayed for hydrolysis of the VSG GPI-anchor. (A) Expression level of GPI-PLC-eYFP wild type and mutant transgenes detected by Western blots probed with anti-GPI-PLC or anti-DHH1 as a loading control. 2×10^6^ cell equivalents were loaded in each track. (B) Western blot lysates of cells expressing GPI-PLC wild type and mutants before and after 20 min incubation in 0.5% triton X-100. The GPI-PLC activity was detected by probing with anti-CRD and anti-BiP was used as a loading control. Two clones are shown for each transgene. (C) One clone expressing each transgene was lysed hypotonically and incubated for 20 minutes at 37°C. Pellet (P) and supernatant (S) fractions were separated at 0 and 20 minutes and analysed by Coomassie staining gels. The white arrows indicate the VSG released. 2×10^6^ cell equivalents were loaded in each track.

Each of the three cysteines in the CCGAC motif was mutated to serine both individually and in combination to make a total of seven mutants. The wild type and each mutant were expressed as a *GPI-PLC-eYFP* transgene from the endogenous locus in a −/− background [37]; therefore any GPI-PLC activity originated from the transgene. These transgenes used the endogenous 3′ untranslated region and resulted in expression levels lower than wild type cells (Figure S1B). The insertion of the transgene had no obvious effect on growth or morphology (data not shown). All the GPI-PLC-eYFP variants had the expected relative molecular mass and similar expression levels (Figure 3A). Wild type and the single cysteine mutants appeared as two polypeptides with slightly different electrophoretic mobilities, whereas the double and triple cysteine mutants appeared homogeneous and with the same mobility as the faster migrating form of the wild type and the single cysteine mutants (Figure 3A). The presence of fatty acids can reduce the rate of protein migration during SDS-PAGE [43] and the slower migrating form might represent more acylated variants. The doublet has been observed previously in both native [14] [44] and recombinant [45] GPI-PLC protein.

### Activity and access to VSG GPI-anchors

The activity of the wild type and each mutant was examined using the detergent lysis assay (Figure 3B). Two independent clones were analysed for each mutant and samples collected 20 minutes after addition of detergent. This time point was chosen as it was close to the end point of hydrolysis in a cell line with a single copy of wild type GPI-PLC (15 to 20 minutes as shown in Figure 1B). The wild type and all the mutants were active as the CRD epitope was produced, indicating that the cysteine motif was not required for GPI-PLC activity. These data confirmed earlier work showing no individual cysteine was necessary for activity in the recombinant protein [45].

Next, the ability of each mutant to access plasma membrane VSG was determined using the hypotonic lysis assay (Figure 3C). One clone of each cell line was examined and samples collected 20 minutes after hypotonic lysis. The vast majority of VSG was released from cells expressing wild type or any of the three single cysteine mutants, indicating that the mutants were able to access VSG GPI-anchors on hypotonic lysis. Only a fraction of the VSG was released from one double cysteine mutant, CSGAS, the remainder pelleting with the cell bodies. Little or no VSG was released on hypotonic lysis of either of the two other double cysteine mutants or the triple cysteine mutant. These data indicate that the cysteine motif has a role in the ability of GPI-PLC to access the VSG GPI-anchors on hypotonic lysis and gives a more precise definition of the requirement than earlier work that showed that all three cysteines are required [18].

### Differential localisation of GPI-PLC-eYFP mutants

**Figure 4:**
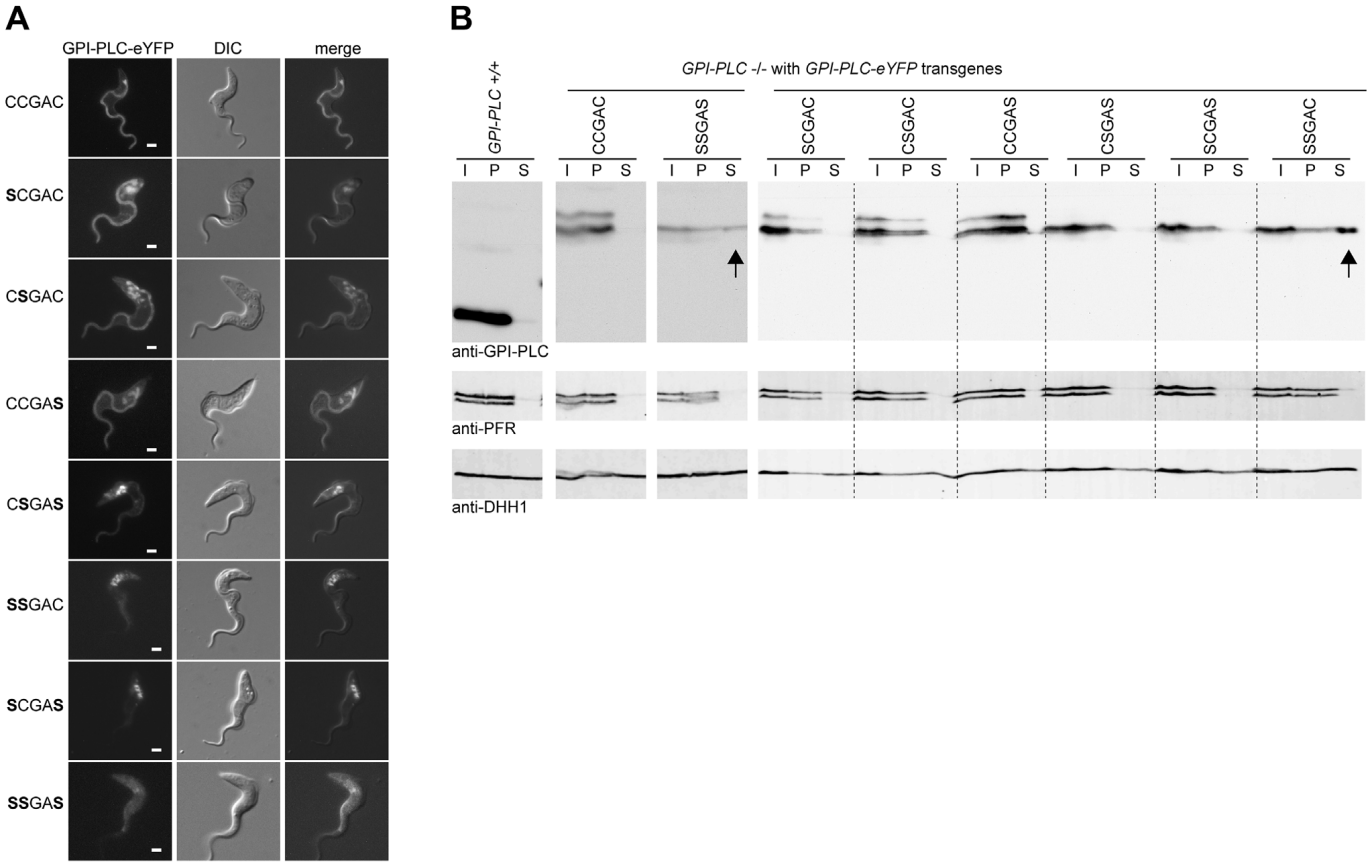
Cysteine mutants mis-localise to the endosome and/or cytoplasm. (A) Cell lines modified to contain a single copy of the wild type (CCGAC) or cysteine mutant *GPI-PLC* all modified with a C-terminal eYFP tag were imaged using the native fluorescence of the eYFP tag after being immobilised. Each image is representative of the distribution observed in the majority of cells. Scale bar represents 2 µm. (B) Wild type (GPI-PLC +/+) and the same cell lines as (A) were hypotonically lysed for 5 minutes on ice and the lysate (I) separated into supernatant (S) and pellet (P) fractions and analysed for the distribution of GPI-PLC-eYFP by Western blotting. The detection of PFR is a control for the cytoskeleton present in the pellet and DHH1 for cytoplasmic proteins. The arrows indicate the presence of GPI-PLC-eYFP in the supernatant fraction. 2×10^6^ cell equivalents were loaded in each track and the blots were cut for presentation purposes.

The inability of some cysteine mutants to hydrolyse the VSG anchor was investigated further by examining the subcellular localisation of GPI-PLC-eYFP variants by fluorescence microscopy (Figure 4A). The wild type was localised to the plasma membrane, and was more concentrated on the flagellar and FP membranes than on the membrane of the cell body. The same localisation was observed when the eYFP tag was located at the N-terminus (Figure S2). This differs from the previously reported localisation to a narrow stripe on the outside of the flagellum membrane between the FAZ and the paraflagellar rod [20]; the possible reasons for the discrepancy are discussed below.

The three single cysteine mutants also localised to the cell membrane in a similar pattern to the wild type with concentration along the entire length of the flagellar membrane. In addition to the plasma membrane localisation, the GPI-PLC variants were also present in the endomembrane system located between the nucleus and flagellar pocket in a fraction of cells. Wild type GPI-PLC-eYFP was associated with these membranes in 8/28 (29%) cells, and the amount detected was low (Table 1). In cells expressing the single cysteine mutants SCGAC and CCGAS, all the cells examined (n = 20, n = 21 respectively) had GPI-PLC-eYFP associated with the endomembranes. In addition, the signal in the mutants was stronger than in the wild type suggesting a larger fraction of the total was membrane associated. In cells expressing the single cysteine mutant CSGAC, 7/22 (32%) contained GPI-PLC-eYFP associated with the same internal membranes, but again the endomembrane signal was stronger than in wild type cells. In the case of the wild type, there was no obvious morphological difference, such as cell cycle stage, between cells with and without internal membrane localised GPI-PLC-eYFP.

**Table 1:** Summary of subcellular localisation and GPI-anchor hydrolysis for wild type and mutant GPI-PLC (n.d - no data).

Motif	Cell and flagellar membrane localisation	Percentage of cells with GPI-PLC associated with the endosomal system (%)	Release of soluble VSG on hypotonic lysis	Mean ratio of GPI-PLC on the flagellar membrane to the cell membrane (±sem)
CCGAC	Y	29	Y	3.8 (±0.3)
SCGAC	Y	100	Y	1.2 (±0.1)
CSGAC	Y	32	Y	2.0 (±0.2)
CCGAS	Y	100	Y	2.1 (±0.3)
CSGAS	Y	100	partial	n.d
SSGAC	N	100	N	n.d
SCGAS	N	100	N	n.d
SSGAS	N	0	N	n.d

The GPI-PLC-eYFP signal intensity from the flagellum was quantified to assess the impact of the mutations. The ratio of fluorescence intensity between a region of the flagellum that had extended beyond the cell body and a region of the anterior cell body was determined for the variants (Figure S3); this ratio provides a semi-quantitative measure of relative abundance. The ratio of intensity (flagellum/cell body) in cells expressing wild type GPI-PLC-eYFP was 3.8±0.3 (±s.e.m) (n = 28). This was reduced in the mutants: SCGAC 1.2±0.1 (n = 20), CSGAC 2.0±0.2 (n = 22) and CCGAS 2.1±0.3 (n = 21). Thus, loss of one cysteine within the motif diminished the ability of GPI-PLC to concentrate on the flagellar plasma membrane and increased the size of the pool present on internal membranes lying between the flagellar pocket and nucleus.

In all cells (n>40) expressing one of the double cysteine mutants, CSGAS, GPI-PLC-eYFP was localised to the cell and flagellar membranes but to a greatly reduced level compared to the wild type and single cysteine mutants: the majority was associated with the endomembrane system (Figure 4). In all cells (n>40) expressing the two other double cysteine mutants, SCGAS and SSGAC, GPI-PLC-eYFP localised predominantly to the same endomembrane system with a fraction present in the cytoplasm (Figure 4A). In all cells (n>40) expressing the triple cysteine mutant SSGAS, there was no longer any obvious membrane association and the protein appeared to be cytoplasmic (Figure 4). To test this further, rapid hypotonic cell lysis was performed, the lysate was separated into a soluble and pellet fraction and the components present in each fraction were analysed by Western blotting using the GPI-PLC antiserum (Figure 4B). In this type of experiment, some cytoplasmic components will be trapped inside cell ghosts but all membrane and cytoskeletal components will be in the pellet. The cytoplasmic protein DHH1 was present in similar amounts in the supernatant and pellet fractions whereas the paraflagellar rod proteins PFR1 and PFR2 were present solely in the pellet. All forms of GPI-PLC-eYFP were present solely in the pellet with the exceptions of the SSGAC and SSGAS mutants, which were distributed in a similar manner to DHH1, providing evidence that a significant fraction was not membrane attached. These data show that the cysteine motif, CCGAC, is necessary for membrane association and has a role in trafficking of GPI-PLC to the cell membrane (summarised in Table 1).

The localisation of some cysteine mutants of GPI-PLC-eYFP to structures lying between the nucleus and flagellar pocket is consistent with association to one or more of the secretory, recycling and lysosomal endosomal compartments. Clathrin localises across a substantial part of the endosomal system [46] and a cell line was made expressing both GPI-PLC-eYFP and a clathrin light chain with a double tomato fluorescent protein tag at the N-terminus (dTomFP-CLC). The dTomFP-CLC was expressed in the GPI-PLC-eYFP double cysteine mutant cell lines (Figure S4). The GPI-PLC-eYFP double cysteine mutants were localised predominantly in the posterior of the cell and partially co-localised with the dTomFP-CLC, indicating that a proportion of the mutant proteins were associated with these endosomal systems. However the co-localisation was not complete and there were regions of GPI-PLC-eYFP signal without a corresponding dTomFP-CLC signal and vice versa, so a definite conclusion about the precise compartment(s) occupied by the internal GPI-PLC could not be drawn.

### The CCGAC cysteine motif is not sufficient for flagellar concentration of GPI-PLC

*Trypanosoma congolense* is the species most closely related to *T. brucei* for which a genome sequence is available. The amino acid sequence of the *T. congolense* GPI-PLC (TcGPI-PLC) has 58% identity with the *T. brucei* GPI-PLC (TbGPI-PLC) (Figure S5), and the conserved residues include the three cysteine motif. To determine whether the targeting signals were conserved, a transgene encoding TcGPI-PLC with a C-terminal eYFP tag (TcGPI-PLC-eYFP) was introduced into a *T. brucei GPI-PLC −/−* cell line. Expression was analysed by Western blotting using a GFP antibody (Figure S6A), Expression was readily detected but the level of TcGPI-PLC was lower than the equivalent cell line containing a TbGPI-PLC-eYFP transgene. Next, the activity of the TcGPI-PLC against the VSG GPI-anchor in two independent clones was analysed using the detergent and hypotonic lysis assays (Figure S6B). On detergent lysis there was a faint CRD signal detected from both clones, indicating that VSG with a hydrolysed GPI-anchor was produced. On hypotonic lysis there was partial release of VSG into the supernatant fraction. These results indicate that TcGPI-PLC was active against the *T. brucei* VSG GPI-anchor and was able to access the GPI-anchors on hypotonic lysis. However, TcGPI-PLC was not as active as TbGPI-PLC; it is unclear whether this was due to less activity against the *T. brucei* VSG GPI-anchor and/or lower expression.

**Figure 5:**
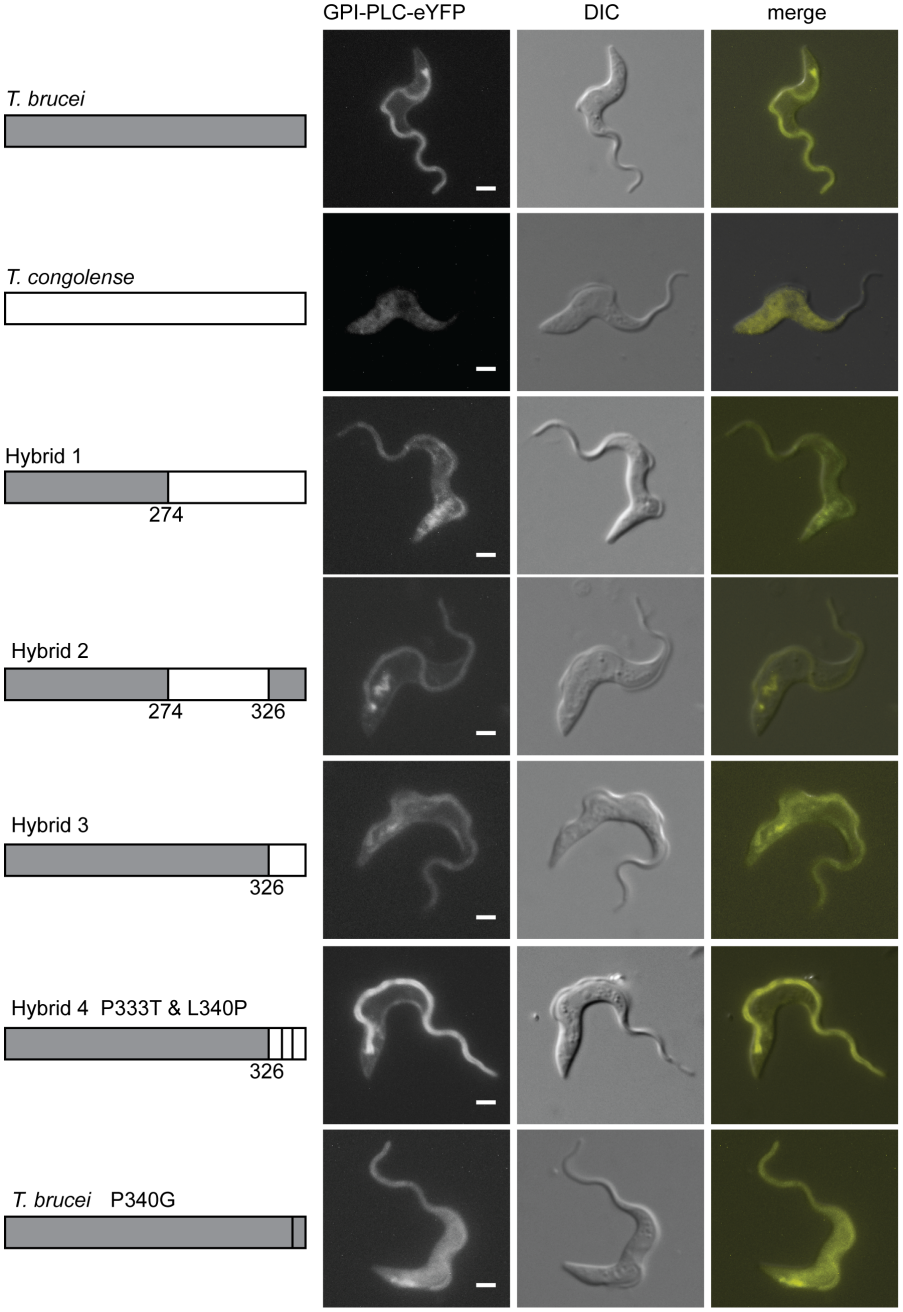
The C-terminal region of GPI-PLC contains a flagellar concentration signal. Cell lines modified to contain a single copy of either wild type *T. brucei* or *T. congolense* GPI-PLC, hybrids or the P340G mutant, all modified with a C-terminal eYFP tag, were imaged using the native fluorescence of the eYFP tag after being immobilised. Each image is representative of the distribution observed in the majority of cells. Scale bar represents 2 µm. The schematic on the left shows the hybrids expressed with the *T. brucei* sequence in grey and *the T. congolense* sequence in white. The position of the switch in sequence is shown above the hybrid sequence and the single residue mutations are indicated. The exposure time used to image the *T. congolense* GPI-PLC was greater than the exposure time for the other cell lines due to the low expression level. Scale bar represents 2 µm.

The localisation of TcGPI-PLC-eYFP was examined by fluorescence microscopy (Figure 5): it was present in the cytoplasm with a fraction associated with the cell membrane as there was a sharp line defining the shape of the cell. TcGPI-PLC-eYFP was not observed on the flagellum.

### Identification of a flagellar membrane concentration signal

As TcGPI-PLC-eYFP did not localise to the flagellum, a flagellar concentration signal was hypothesised to be located in the regions of TbGPI-PLC that did not share identity with TcGPI-PLC. To test this theory, a series of TbGPI-PLC and TcGPI-PLC hybrids was constructed (Figure S7 and S5). The points of fusion were located in regions of identity between TbGPI-PLC and TcGPI-PLC and the hybrids were expressed with an eYFP tag at the C-terminus in the *GPI-PLC −/−* cell line.

The expression of the hybrids was analysed by Western blotting with a GFP antibody (Figure S6A): all were the expected relative molecular mass and the expression levels were all similar to that of wild type TbGPI-PLC-eYFP. The localisation of the hybrids was examined by fluorescence microscopy (Figure 5). Hybrid 1 consisted of the N-terminal region of TbGPI-PLC up to the CCGAC motif, after which the TcGPI-PLC sequence was present. Hybrid 1 localised to the cell and flagellar membranes but there was also a large proportion associated with the endomembrane system and possibly some present in the cytoplasm (Figure 5). In order to quantify the effects of the different hybrids on GPI-PLC localisation, the ratio of the fluorescence intensity from a region of the flagellum away from the cell body to the fluorescence intensity of a region of the anterior cell body was calculated as above. Wild type TbGPI-PLC had a ratio of 3.8±0.3 (±s.e.m) (n = 28); hybrid 1 had a ratio of 1.0±0.1 (n = 40). The N-terminal part of TbGPI-PLC up to and including the CCGAC motif was able to support membrane localisation but hybrid 1 did not concentrate in the flagellum.

Hybrid 2 was derived from hybrid 1 by replacing the C-terminal 32 with the equivalent region from TbGPI-PLC. Hybrid 2 localised to both the cell and flagellar membranes and a fraction associated with the endomembrane system, and the overall appearance of the cells was similar to that of the wild type TbGPI-PLC (Figure 5). However for hybrid 2 the ratio of fluorescence intensity was 1.2±0.1 (n = 14), only marginally higher than that for hybrid 1. Hybrid 3 consisted of TbGPI-PLC but with the C-terminal 32 residues replaced with the equivalent sequence from TcGPI-PLC. Hybrid 3 localised to the cell and flagellar membranes with a large proportion associated with the endomembrane system (Figure 5) and a fluorescence intensity ratio of 1.0±0.1 (n = 23). Hybrid 3 was similar to hybrid 1 in terms of its localisation pattern and fluorescence intensity ratio, suggesting that the C-terminal 32 residues were necessary for the concentration of TbGPI-PLC on the flagellar membrane.

The C-terminal 32 residues of TbGPI-PLC and TcGPI-PLC are similar with 50% identity. One of the differences is the location of a proline residue: the C-terminal 32 residues from both proteins contain a single proline but the location is not conserved (Figure S5). Hybrid 4 was derived from hybrid 3: it contained the C-terminal 32 residues from TcGPI-PLC altered to move the single proline to the equivalent position in TbGPI-PLC (P333T and L340P). Hybrid 4 localised to both the cell and flagellar membranes and was concentrated on the flagellar membrane (Figure 5). The fluorescence intensity ratio for hybrid 4 was 1.9±0.2 (n = 13), an increase in the concentration on the flagellar membrane compared to hybrid 3. To determine whether the P340 was necessary for concentration on the flagellar membrane, a point mutation of P340G in TbGPI-PLC was tested. The mutation resulted in a dramatic change in the localisation: GPI-PLC-eYFP P340G was evenly distributed over the cell and flagellar membranes, which was confirmed by the drop in the fluorescence intensity ratio to 0.4±0.03 (n = 10) (Figure 5). This result showed that P340 was necessary for the flagellar membrane concentration of TbGPI-PLC but not for association with the plasma membrane. Two independent clones expressing GPI-PLC-eYFP P340G were assayed for VSG release after hypotonic lysis (Figure S8). The mutant was able to release VSG on hypotonic lysis, indicating that it was active and able to access its substrate.

Further mutants designed to determine the full extent of the motif containing P340 were inconclusive. GPI-PLC mutants containing alterations to the C-terminal side of P340 (M341A and 341 MNAV to 341 AAAA) were able to concentrate on the flagellar membrane (Fig. S9). A mutation to the N-terminal side (K339A) destabilised the GPI-PLC-eYFP to the extent that localisation data was not obtained. The motif causing flagellar concentration does not extend to the C-terminal side of P340 but may extend to the N-terminal side.

### Loss of the FPC and FP causes a redistribution of GPI-PLC

Little is known about how proteins are concentrated in different domains of the trypanosome plasma membrane. Components from the structures that are believed to delimit the different domains have been identified, such as BILBO1, a component of the FPC, located at the junction of the cell body and FP plasma membrane domains. In proliferating cells, RNAi knockdown of BILBO1 results in a failure to form the new FPC and FP. In addition, the new flagellum is detached from the cell body and migrates to the posterior of the cell [24]. A feature of the trypanosome flagellum is that it is attached to the cell body as far as the anterior pole of the cell and then extends beyond the cell body. Components of the FAZ are necessary for the attachment: RNAi knockdown of FAZ components results in the newly synthesised flagellum being detached from the cell body, but the formation of the FP is unaffected. FLA3 is a FAZ component upregulated in bloodstream form trypanosomes and RNAi knockdown causes the new flagellum to be synthesised without attachment to the cell body but, importantly, there is no apparent effect on the FPC or FP [47].

Cell lines were constructed expressing a GPI-PLC-eYFP transgene and containing plasmids to direct tetracycline-inducible RNAi against BILBO1 or FLA3. The population doubling times of the BILBO1 and FLA3 RNAi cells were measured as 12 and 7 hours respectively. RNAi was then induced for a single doubling time before the localisation of GPI-PLC-eYFP was determined. As the induced cells had undergone one cell cycle, the majority of the population of cells had one old flagellum, constructed before induction of RNAi, and one new flagellum displaying the RNAi phenotype. This time point allowed any changes in GPI-PLC localisation caused by the RNAi to be detected by comparing the new and old flagella.

**Figure 6:**
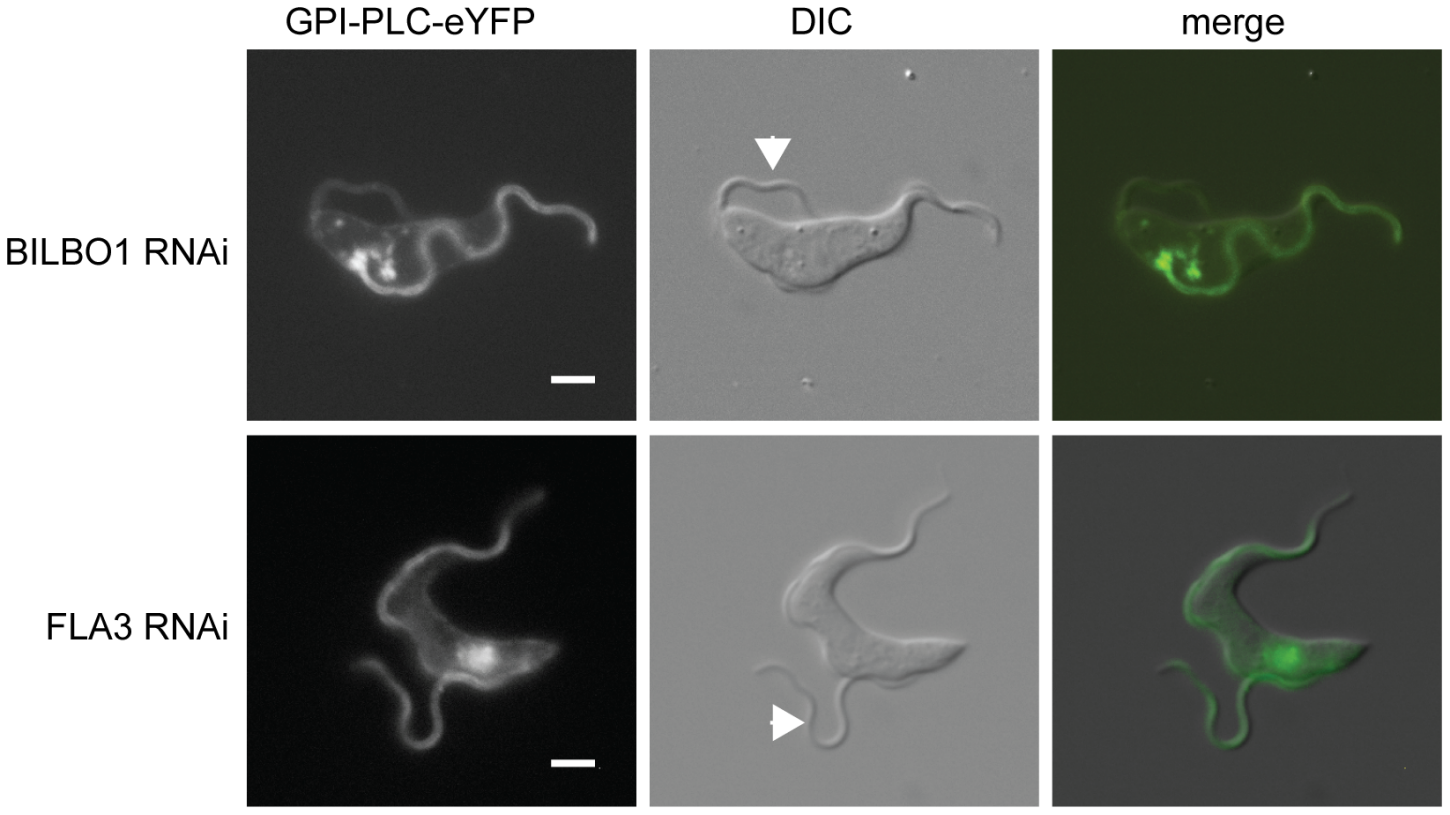
An intact FPC is necessary for the maintenance of GPI-PLC flagellar concentration. The ‘single marker’ bloodstream form RNAi cell line was modified to contain a *GPI-PLC-eYFP* transgene at the endogenous locus and then modified FPC component BILBO1 or the FAZ component FLA3. The images are representative of the native fluorescence from the eYFP in immobilised post-induction cells with one attached (old) and one detached (new) flagellum. The arrowheads indicate the new flagellum. Scale bar represents 2 µm.

On induction of BILBO1 RNAi, the new flagellum was detached and had migrated to the posterior pole of the cell in the majority of cells as described previously [25]. In these cells, GPI-PLC-eYFP localised to the plasma membrane (Figure 6A); however, there was a reduction in GPI-PLC-eYFP concentration in the new flagellum and in some cells there was no difference between the intensity of GPI-PLC-eYFP in the new flagellum and the intensity on the cell body. In all the cells examined, the old flagellum retained a higher concentration of GPI-PLC-eYFP than the plasma membrane, as might be predicted since the loss of BILBO1 does not affect the existing FPC.

The localisation of GPI-PLC-eYFP after induction of FLA3 RNAi is shown in Figure 6B. Cells were examined that had an attached old flagellum and a detached new flagellum. FLA3 knockdown had no apparent effect on GPI-PLC-eYFP localisation: GPI-PLC-eYFP localised to the plasma membrane and was concentrated to a similar extent in both the old and new flagella.

To quantify the effect of BILBO1 or FLA3 knockdown on the ability of GPI-PLC-eYFP to concentrate in the detached new flagellum, the ratio of the GPI-PLC-eYFP fluorescence intensity between a region of the attached old flagellum that extended beyond the cell body and the detached new flagellum was calculated; both values had the background fluorescence intensity subtracted before the ratio was calculated (Figure 6). A value close to 1 would be expected, as the old and new flagella should contain the same set of proteins at similar levels. The ratio for the FLA3 knockdown cells was 1.2±0.1 (±s.e.m) (n = 8), implying that the detachment of the flagellum by FLA3 knockdown does not greatly affect the ability of GPI-PLC to concentrate in the detached new flagellum. For BILBO1 knockdown cells, the ratio was 2.2±0.3 (n = 18). This value was significantly different (p<0.005) to that found for the FLA3 knockdown, indicating that there was a loss of GPI-PLC-eYFP flagellar concentration on BILBO1 knockdown. Furthermore, the defect in GPI-PLC-eYFP flagellar concentration appeared to be related to the loss of the FPC and/or FP and not to the detachment of the flagellum, as there was no apparent loss of GPI-PLC-eYFP concentration in the flagellum on FLA3 knockdown.

## Discussion

Bloodstream form trypanosomes contain sufficient GPI-PLC to release the entire VSG coat within a few minutes, yet this release only occurs after the integrity of the plasma membrane has been compromised. Previous work on the regulation of GPI-PLC activity has shown that there is dynamic acylation [19] and that three cysteines present in the CCGAC motif are necessary for acylation and access to VSG substrate in ruptured cells [18]. The latter suggested that localisation to membranes was necessary for access to the VSG and was consistent with the subsequent finding that GPI-PLC is concentrated on the flagellar membrane [20]. Here, the determinants of this localisation have been identified and the effect of localisation on access to VSG substrate determined. The major findings are: (i) the cysteines in the CCGAC motif act in a dose-dependent manner in determining localisation to the plasma membrane; (ii) association of the GPI-PLC with the plasma membrane is necessary for VSG release on hypotonic lysis, association with the endomembrane system between the nucleus and flagellar pocket is not sufficient; (iii) a proline, residue 340, close to the C-terminus is necessary for concentration of GPI-PLC on the flagellar membrane; (iv) GPI-PLC does not concentrate on a flagellum without a flagellar pocket collar and/or flagellar pocket.

The CCGAC motif is acylated [18], [19] and the cysteines are required for complete localisation to the plasma membrane (Figure 4). Once associated with the plasma membrane, GPI-PLC is predominantly on the cytoplasmic face as shown by the lack of proteolysis of GPI-PLC in live cells when treated with a high external concentration of trypsin (Figure 2). The reason for the discrepancy between this finding and the earlier finding that GPI-PLC is localised on the external face of the plasma [20] probably lies in the assay used. Here, trypsin access to the GPI-PLC in live cells assays for all the GPI-PLC in the cell, whereas the surface labelling used previously [20] did not as the fraction labelled was not determined. In addition Bülow and colleagues analysed the sensitivity of GPI-PLC activity to trypsin and found that its activity was insensitive to trypsin until detergent was added to the cells [48]. It is also worth noting that cytoplasmically acylated proteins have, to date, always been reported to be associated with the cytoplasmic face of membranes [23], [49], [50], [51], [52], [53].

The subcellular localisation of GPI-PLC-eYFP to the plasma membrane and concentration on the flagellar membrane is distinct from that idenitified by previous studies which have used immunofluorescence and other assays to propose a range of localisations; the list is described and discussed in [20]. There is only one report [20] that demonstrated monospecificity of the antibody by Western blotting and specificity in immunofluorescence using *GPI-PLC −/−* cells. Deconvolution of confocal images of immunofluorescence experiments was used to report a localisation to a narrow stripe on the flagellar membrane near the FAZ. Here, the localisation of GPI-PLC-eYFP reported has a wider distribution, encompassing the whole plasma membrane, with concentration on the flagellar membrane. Why is there a discrepancy between the results here and the earlier report [20]? There is a range of possibilities; the first is that the use of the eYFP tag alters the localisation. This must remain a possibility although it is unlikely as the same localisation was obtained with the eYFP at the N- and C-termini (Figure S2). An alternative is that the fixation used prior to the immunofluorescence-determined subcellular localisation restricted the access of the antibodies to a subset of the GPI-PLC and it is worth noting that fixation conditions did affect the results [20]. Further, there is a history of contradictory results on the subcellular localisation of the GPI-PLC determined by immunofluorescence suggesting that access to and/or detection of GPI-PLC is not facile. A second possibility for the difference is the deconvolution of the images that resulted in the original localisation of GPI-PLC-eYFP to the flagellum [20]. The deconvolution process removed all but the strongest fluorescent signal for GPI-PLC-eYFP as if the remainder were out-of-focus fluorescence and producing a bias towards a discrete structure. The result was an artefactual localisation to the region where GPI-PLC-eYFP was most concentrated, the flagellar membrane. To illustrate this point, a comparison PFR1-mCherryFP and GPI-PLC-eYFP localisation shows the dispersion of GPI-PLC-eYFP to the plasma membrane compared to the discrete localisation of PFR1 to the paraflagellar rod (Figure S10).

The number of cysteine residues affected the mobility of GPI-PLC on SDS-PAGE. Forms with two or more cysteines appeared as a doublet whereas forms with one cysteine or none appeared as a single band, equivalent to the faster migrating form. This observation provides evidence that the doublet results from a mixture of differently acylated forms present in the cell at time of lysis and that the slower migrating band is multiply acylated. The number of acylated residues within the cysteine motif has not been quantified but as the loss of one cysteine residue reduces the flagellar concentration of GPI-PLC it appears that all three have to be modified for correct localisation. Acylation of GPI-PLC is a dynamic process and occurs in cells where protein synthesis has been inhibited with cycloheximide. It is likely that the acylation patterning on GPI-PLC can be continuously remodelled [19]. The ability to remodel the modifications on the protein is similarly found for palmitoylated proteins in other systems, such as SNAP25 in mammalian cells [54]. The identity of the acyl transferase acting on GPI-PLC is unknown: there are >10 in the trypanosome genome [23]. However, the recombinant protein produced in *E. coli* also runs as a doublet on SDS-PAGE [45], which suggests that either the GPI-PLC is a substrate for a bacterial non-specific acyl transferase or that the acylation is spontaneous. In either case, no great specificity is required to acylate GPI-PLC.

The cysteine motif CCGAC had previously been implicated in the regulation of the activity and localisation of GPI-PLC but the role of individual cysteines within the motif had not been studied [18]. All mutants retained activity for VSG GPI-anchor hydrolysis on detergent lysis of cells. Thus, acylation of the CCGAC motif is not necessary for activity in vitro and no large reduction in activity was visible in the absence of acylation. In contrast, the ability to access the VSG substrate on hypotonic lysis was strictly dependent on localisation. When a single cysteine was mutated there was a decrease in the concentration of GPI-PLC within the flagellar membrane and an increase in the association of GPI-PLC with the endomembrane system. However, there was no observable effect on access to VSG substrate on hypotonic lysis. When two cysteines were mutated, there was a dramatic redistribution of GPI-PLC from the flagellar membrane to the endomembrane system. There was also an effect on the ability of the mutants to access the VSG on hypotonic lysis. With the CCGAC→CSGAS mutant, there was only partial release of VSG and the SSGAC and SCGAS mutant were unable to release VSG from the plasma membrane. The mutation of all three cysteines caused the GPI-PLC to localise to the cytoplasm and it was unable to access the GPI-anchor on hypotonic lysis. These data provide evidence that GPI-PLC is only able to access the GPI-anchors on hypotonic lysis when localised to the plasma membrane and that the plasma membrane is the site of action of GPI-PLC. The concentration of GPI-PLC on the flagellar membrane was not required for the release of VSG on hypotonic lysis as the GPI-PLC P340G mutant was able to hydrolyse the VSG GPI-anchors, despite the mutation resulting in the redistribution of GPI-PLC. These observations are consistent with an earlier model in which osmotic rupture of the cells allowed VSG and GPI-PLC to mix through passive diffusion around the edges of discontinuities in the plasma membrane [35]. Since the VSG and GPI-PLC have to be in the same membrane for hydrolysis to occur [8] any vesicles derived from endosomes or free GPI-PLC in the cytoplasm presumably cannot access VSG [34]. The simplest model for GPI-PLC regulation is that it cannot access the VSG substrate in live cells as it is on the opposite side of the membrane. In proliferating bloodstream form trypanosomes, the half-life of the VSG is several cell generations [12], [13] and thus this regulation is very effective. However, the VSG is shed during differentiation from bloodstream forms to insect infective procyclic forms. During the process, both GPI-anchor hydrolysis and proteolytic cleavage by MSP-B, a cell surface metalloprotease, result in VSG release: either enzyme is sufficient to complete the process [55]. This means that the GPI-PLC has regulated access to the VSG during the differentiation process in cells that remain viable and go on to divide. The mechanism remains to be investigated but might provide an explanation for the concentration of the GPI-PLC on the flagellum.

In addition to acylation, proline 340 is necessary for concentration of the GPI-PLC on the flagellar membrane. GPI-PLC from the closely related *T. congolense* (TcGPI-PLC) has 58% identity with the *T. brucei* GPI-PLC (TbGPI-PLC), including the cysteine motif. However, in *T. brucei* the localisation of TcGPI-PLC appeared to be mainly cytoplasmic with a small proportion localised to the cell membrane. TcGPI-PLC did not have the same pattern of localisation as TbGPI-PLC despite the presence of the cysteine motif, suggesting that the cysteine motif is not sufficient for the concentration of GPI-PLC within the flagellum. Other proteins that rely on acylation for flagellar localisation, such as rhodopsin, also have another flagellar localisation signal [28]. Therefore a flagellar concentration signal was hypothesised to be located in the regions of the TbGPI-PLC sequence that did not share identity with the TcGPI-PLC sequence (Figure S4). A single proline residue with this C-terminal region was shown to be a key GPI-PLC flagellar concentration signal and its mutation resulted in GPI-PLC failing to concentrate on the flagellar membrane. Two other flagellar membrane localisation motifs have been described in kinetoplastids. The flagellar calcium binding protein from *Trypanosoma cruzi* is myristoylated and palmitoylated at the N-terminus. These acylations are necessary but not sufficient for flagellar localisation, which also requires three lysine residues located between residues 13 and 22 [56]. Furthermore, residues 1 to 24 were sufficient for localisation of a GFP reporter to the flagellar membrane and this same reporter was localised to the flagellum in *Leishmania amazonensis* suggesting a conserved mechanism. The second motif was identified in the flagellar glucose transporter 1 from *Leishmania mexicana* (LmxGT1; LmxM.36.6300) [57]. LmxGT1 contains 12 membrane-spanning segments located after residue 131; the N-terminal 130 residues are present in the cytoplasm. A mutational analysis of the N-terminal 130 residues to locate sequences necessary for flagellar localisation identified a motif between residues 95–97 with the sequence asn-pro-met (NPM). This is similar to the context of P340 in GPI-PLC lys-pro-met (KPM), and it is possible that LmxGT1 and GPI-PLC are substrates for the same flagellar concentration mechanism. The proline may be part of a motif or may act as a substrate for a peptidyl prolyl isomerase that regulates accumulation on the flagellar membrane. The isomerisation of the proline could re-orient the structure of the GPI-PLC at the C-terminus; the rearranged C-terminus may then be able to bind to another factor that enables flagellar concentration. Proteomic analysis has identified peptidyl prolyl isomerases within the trypanosome flagellum so the appropriate enzymes are present [58].

There are minimally three domains in the plasma membrane in trypanosomes: the cell body, the flagellar pocket and the flagellum. The FPC is a protein ring structure located at the point at which the flagellar pocket invaginates from the cell body membrane and is necessary for the formation of a FP. BILBO1 was identified as a component of the FPC, which maintains the structure of the FP [25]. On BILBO1 knockdown by RNAi there is loss of the new FP and new FPC, and detachment of the new flagellum. The knockdown affected GPI-PLC localisation: it was no longer concentrated in the new flagellum. In order to determine if the loss of GPI-PLC concentration was due to the loss of the FPC and/or FP or flagellar attachment, a second RNAi was carried out that targeted FLA3. RNAi knockdown of FLA3 results in the detachment of the new flagellum but importantly the FPC remains intact [47]. The mean ratio of GPI-PLC concentration between the old and new flagella in the FLA3 knockdown cells was approximately 1, which would be expected, as the new and old flagella should contain a similar set of proteins; the detachment of the flagellum therefore does not affect the localisation of GPI-PLC. In the absence of a FPC and/or FP there is no concentration of GPI-PLC on the flagellum but it is not possible to distinguish whether flagellar concentration is a direct function of the FPC or the FP as both are lost on BILBO1 knockdown. The observations do provide evidence for the long-held view that there are selective diffusion barriers for proteins at the boundaries between the plasma membrane domains in trypanosomes. A flagellum-associated diffusion barrier has been demonstrated in other organisms by the knockdown of CEP290, a component of the transition zone in the flagellum of *Chlamydomonas reinhardtii*, and Septin 2, found at the base of mammalian primary cilia [59], [60]. The loss of these proteins led to a redistribution of proteins between the flagellar and cell membrane. However, during BILBO1 knockdown the new FP is also lost: as GPI-PLC is trafficked through the FP, the reduction in GPI-PLC-eYFP concentration within the new flagellum could be due to a lack of GPI-PLC reaching the new flagellum. Although BILBO1 is a key component of the FPC diffusion barrier, which restricts the movement of GPI-PLC and could therefore be responsible for maintaining GPI-PLC concentration within the flagellum, there is no evidence that BILBO1 interacts directly with GPI-PLC.

## Materials and Methods

### Trypanosomes

*Trypanosoma brucei* Lister 427 bloodstream form cells were used in all experiments. All cells were grown in HMI-9 medium with 10% foetal bovine serum. All experiments were performed with logarithmically growing trypanosomes at a cell density of less than 1×10^6^ cells/ml. The *GPI-PLC* −/− cell line has been described [37] and was made by replacing the entire gene at both alleles with selectable marker genes. The transgenic cell lines expressing GPI-PLC variants in the *GPI-PLC −/−* background were made as described [37] by returning a copy of the modified gene, including 5' and 3′ UTRs, to the endogenous locus with expression relying on endogenous transcription. The cell line *GPI-PLC-eYFP/−* was made by modifying the remaining *GPI-PLC* allele in the *GPI-PLC/−* cell line with a C-terminal eYFP tag as described [36]. The cell line expressing both wild type and GPI-PLC-eYFP (*GPI-PLC-eYFP*/+) was made using the same approach except that the transgene was introduced into a GPI-PLC +/+ cell line. The RNAi experiments used the Lister 427 328.114 single marker cell line [38] (a kind gift of George Cross). An endogenous *GPI-PLC* allele in the single marker cell line was tagged at the C-terminus with eYFP as described [36]. The expression of the N-terminal dTomato fluorescent protein tagged clathrin light chain was from a modified endogenous locus and relied on endogenous transcription [36]. Transgenic trypanosomes were generated using standard procedures [61].

### Plasmids

Details of all plasmids are described in Table S1. The sequences of all plasmids are available from the authors.

### Detergent cell lysis

Cells were harvested by centrifugation and washed with 10 ml of HMI-9 without serum and re-centrifuged. The cells were then resuspended in HMI-9 without serum at 1×10^8^ cells/ml, 0.05 volumes of 10% (v/v) Triton X-100 was added and the mixture incubated at room temperature. Samples were removed after 0, 2, 5, 10 and 20 minutes and were analysed by SDS-PAGE and Western blotting.

### Hypotonic cell lysis

Cells were harvested by centrifugation and washed with 10 ml of ice-cold HMI-9 without serum and re-centrifuged. The cells were resuspended in ice-cold 1 mM TLCK at 1×10^8^ cells/ml and incubated on ice for 5 minutes then incubated at 37°C for a further 15 minutes. Samples were removed at 0, 1, 5, 7, 10, 15 and 20 minutes and fractionated into pellet and supernatant fractions by centrifugation in a microfuge, 13,000 rpm for 1 minute. The samples were analysed by SDS PAGE and Western blotting. The GPI-PLC cysteine mutants were subjected to a simplified assay in which the cells were resuspended in 1 mM TLCK at 1×10^8^ cells/ml and incubated for 20 minutes at room temperature followed by SDS PAGE and Western analysis.

### Cell fractionation after hypotonic lysis

Hypotonic lysis was performed as above. After 5 minutes on ice the samples were vortexed for 30 seconds, a sample of the lysate was taken and the remainder separated into the pellet and supernatant fraction by centrifugation in a microfuge 13,000 rpm for 1 minute. The samples were then analysed by Western blotting as above.

### Western blots

Western blots were performed using standard protocols. However a high pH transfer buffer (19 mM glycine, 20 mM Tris base, 0.08% (w/v) SDS and 15% (v/v) methanol) was used to ensure consistent transfer of GPI-PLC as it has an unusually high pI. Detection was by either by a fluorescent secondary antibody using the Odyssey Infrared Imaging System or by enhanced chemiluminescence (ECL).

### Antibodies

Recombinant full length His-tagged GPI-PLC was produced using the baculovirus system. The recombinant protein was purified on a nickel affinity column. The purified protein was run into an SDS-PAGE gel and then electro-eluted from the gel. The eluted protein was used to inoculate a rabbit (Covalab). Recombinant GPI-PLC was blotted onto PVDF and used to affinity purify anti-GPI-PLC antibodies from the rabbit sera. Anti-CRD was prepared by immunising a rabbit with VSG ILTat1.21 and antibodies were affinity purified using immobilised VSG MITat1.2. Other antibodies have been described: ISG65 [62], BiP [63], DHH1 [41], PFR (L13D6) [64]. Anti-GFP was obtained from Invitrogen (A11122).

### Trypsin treatment

Cells were harvested by centrifugation washed with 10 ml of ice-cold HMI-9 without serum and recentrifuged. For live cell trypsin treatment the cells were resuspended in HMI-9 without serum to 4×10^7^ cells/ml and trypsin was added to a final concentration of 40 µg/ml. For lysed cell trypsin treatment the cells were resuspended in HMI-9 without serum, lysed with 0.05 volumes of 10% (v/v) Triton X-100 and trypsin added to a final concentration of 100 µg/ml. The mixture was incubated at room temperature. Samples were removed at 0, 3, 6, 9, 12, 15 and 18 minutes and analysed by SDS-PAGE and Western blotting. ECL was used to detect the GPI-PLC-eYFP signal. The GPI-PLC, ISG65, PFR1, PFR 2 and DHH1 signals were detected using the Odyssey Infrared Imaging System and quantification was carried out using the Odyssey software.

### Microscopy and image analysis

Cells from 1 ml culture were centrifuged at 10000 rpm for 1 minute in a microfuge and washed with 1 ml of HMI-9 without serum. The cells were then centrifuged as before and resuspended in 50 µl of HMI-9 without serum. To immobilize the cells, formaldehyde was added to a final concentration of 0.075% (v/v) for 5 minutes, images were taken within the next 20 minutes. Great care was taken to check the immobilisation with formaldehyde had no visible effect on the distribution of the tagged protein. Cells were visualised using a Zeiss Axioimager M1; images were recorded using the Axiovision software (Zeiss) and then imported into Adobe Photoshop. Fluorescent intensity measurements were performed on individual cells using the ImageJ software (NIH). The fluorescent intensity was measured from an area of the cell body or an area of the flagellum, where the flagellum had extended beyond the cell body, in cells with a single flagellum. The mean fluorescent intensity was taken from these measurements and the background fluorescence subtracted before a ratio of the fluorescent intensities was calculated. The ratios reported are the mean values ± standard error of the mean. An unpaired Student t-test was used to calculate the level of significance between the means.

## Supporting Information

Figure S1 A. Western blot probed with anti-GPI-PLC of *GPI-PLC +/−* and *GPI-PLC-eYFP/−* cell lines. The expression level of the tagged GPI-PLC is similar to the untagged GPI-PLC. A comparison of *GPI-PLC +/+* and *+/−* expression is shown in reference 37. B. Relative expression levels of GPI-PLC and mutants. Western blot analysis of GPI-PLC and GPI-PLC-eYFP in wild type (*GPI-PLC +/+*), null (*GPI-PLC −/−*) and null cells further modified by the introduction of transgenes encoding modified *GPI-PLC* genes. The levels of all GPI-PLC-eYFP variants was below wild type levels. Anti-DHH1 was used as a loading control and 2×10^6^ cell equivalents were loaded per track. The asterisk marks a cross reacting band present in some samples.
(TIF)

Figure S2 Typical images of cells expressing GPI-PLC with a C-terminal or N-terminal eYFP tag. 50 cells with either tag were examined and the localisation of GPI-PLC in all was on the cell membrane with clear concentration on the flagellar membrane. Scale bar represents 2 µm.
(TIF)

Figure S3 Diagram to illustrate the method to measure the fluorescent intensity ratio between the flagellum and the cell body.
(TIF)

Figure S4 Images of representative cells expressing eYFP tagged GPI-PLC double cysteine to serine mutants with dTomatoFP tagged CLC. The CLC marks part of the endosomal system and there was clear overlap with the GPI-PLC cysteine mutants. Scale bar represents 2 µm.
(TIF)

Figure S5 Alignment of GPI-PLC from *T. brucei* and *T. congolense*. The cysteines in the CCGAC motif are shown in red. The proline residues that were mutated towards the C-terminus are also shown in red. The points at which fusion constructs were joined are highlighted in yellow. *T. brucei* numbering is used throughout.
(DOCX)

Figure S6 A) Western blot of cells expressing a variety of eYFP tagged GPI-PLC constructs probed with anti-GFP and anti-DHH1 (loading control). B) Western blot of detergent lysis of cells expressing *T. brucei* GPI-PLC and *T. congolense* GPI-PLC probed with anti-CRD and anti-BiP (loading control). *T. congolense* GPI-PLC was partially active. C) Coomassie stained gel of hypotonic lysis of cells expressing *T. congolense* GPI-PLC. The arrows indicated the sVSG released. P = pellet, S = supernatant.
(TIF)

Figure S7 Schematic of the *T. brucei* and *T. congolense* hybrids constructed. Grey corresponds to *T. brucei* sequence and white corresponds to *T. congolense sequence*. The sequence of the hybrid at the point the sequences switch is shown within the protein and the residue number at which the switch occurs is above the sequence. Mutations are shown above and below the sequence with the original residue, its number and the residue it becomes.
(TIF)

Figure S8 Coomassie stained gels of hypotonic lysis assays of cells expressing GPI-PLC P340G. The arrows indicated the sVSG released. P = pellet, S = supernatant.
(TIF)

Figure S9 Mutations to the C-terminal side of P340 do not affect flagellar concentration of GPI-PLC. The mutations are shown above the representative images, P340 is indicated in red.
(TIF)

Figure S10 Images of typical cells expressing GPI-PLC-eYFP and PFR1-mCherryFP. 200 cells were imaged and all were similar to the cells above. Scale bar represents 2 µm.
(TIF)

Table S1 Table describing the plasmids used in this study. p1906 is described in Webb et al., (2005). p2T7-177 is described in Wickstead et al., (2002). p2937 and p3121 are derivatives of p2710 and p2679 respectively (Kelly at al., 2007): p2937 contains a gene for blasticidin resitance as opposed to the gene for G418 resistance in p2710. p3121 contains an dTomatoFP tag as opposed to the mCherryFP tag in p2679. The base plasmids p3261, p3649 and p3731 were constructed for this study. p3261 is a derivative of p1906 with a BglII site introduced before the stop codon (no change in amino acid sequence) and an a GSGSGS linker followed by eYFP added to the C-terminus. p3649 is a derivative of p3261 with a SmaI site introduced downstream of the cysteine motif (no change in amino acid sequence) to allow introduction of the cysteine motif mutants. p3731 is a derivative of p3649 with a HindIII site introduced before the start codon to allow the introduction of GPI-PLC hybrids as HindIII BamHI fragments into the HindIII BglII sites. The sequences of all plasmids are available from the authors.
(DOC)
