# Supplementary figures and images for: Determinants of GPI-PLC Localisation to the Flagellum and Access to GPI-Anchored Substrates in Trypanosomes
Source: PLoS Pathog. 2013 Aug 22;9(8):e1003566. doi: 10.1371/journal.ppat.1003566 (PMC3749955; doi:10.1371/journal.ppat.1003566)

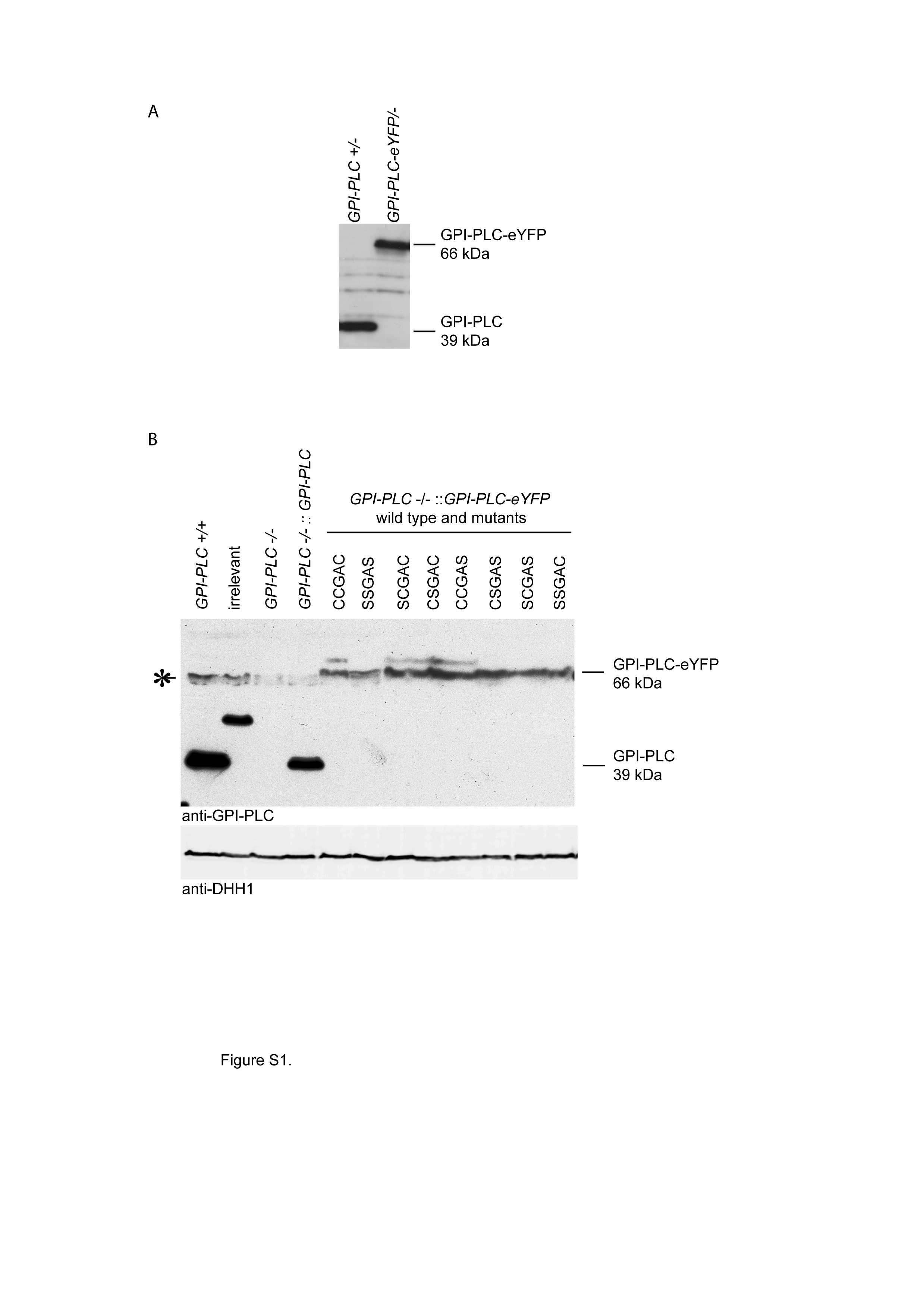

Supplement: Figure S1 — A. Western blot probed with anti-GPI-PLC of GPI-PLC +/− and GPI-PLC-eYFP/− cell lines. The expression level of the tagged GPI-PLC is similar to the untagged GPI-PLC. A comparison of GPI-PLC +/+ and +/− expression is shown in reference 37. B. Relative expression levels of GPI-PLC and mutants. Western blot analysis of GPI-PLC and GPI-PLC-eYFP in wild type (GPI-PLC +/+), null (GPI-PLC −/−) and null cells further modified by the introduction of transgenes encoding modified GPI-PLC genes. The levels of all GPI-PLC-eYFP variants was below wild type levels. Anti-DHH1 was used as a loading control and 2×10^6^ cell equivalents were loaded per track. The asterisk marks a cross reacting band present in some samples. (TIF) [file ppat.1003566.s001.tif]

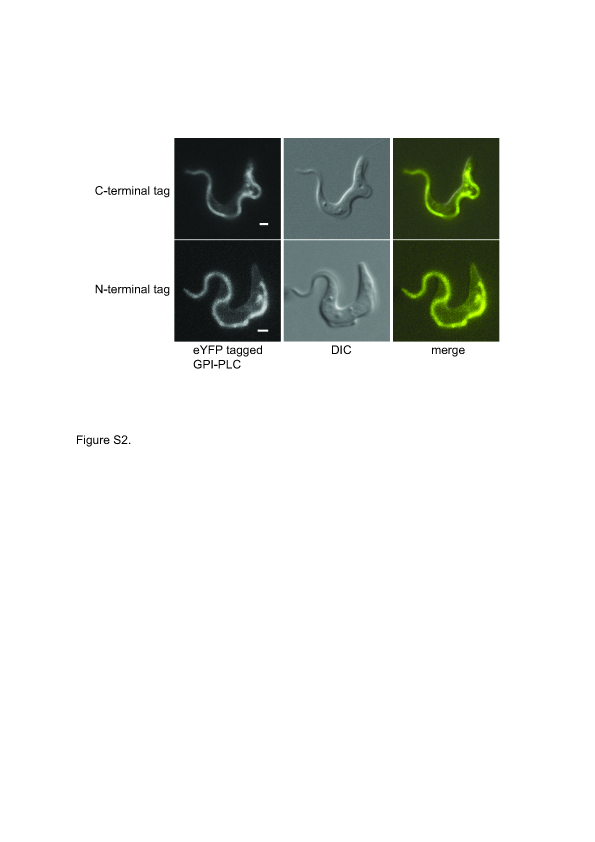

Supplement: Figure S2 — Typical images of cells expressing GPI-PLC with a C-terminal or N-terminal eYFP tag. 50 cells with either tag were examined and the localisation of GPI-PLC in all was on the cell membrane with clear concentration on the flagellar membrane. Scale bar represents 2 µm. (TIF) [file ppat.1003566.s002.tif]

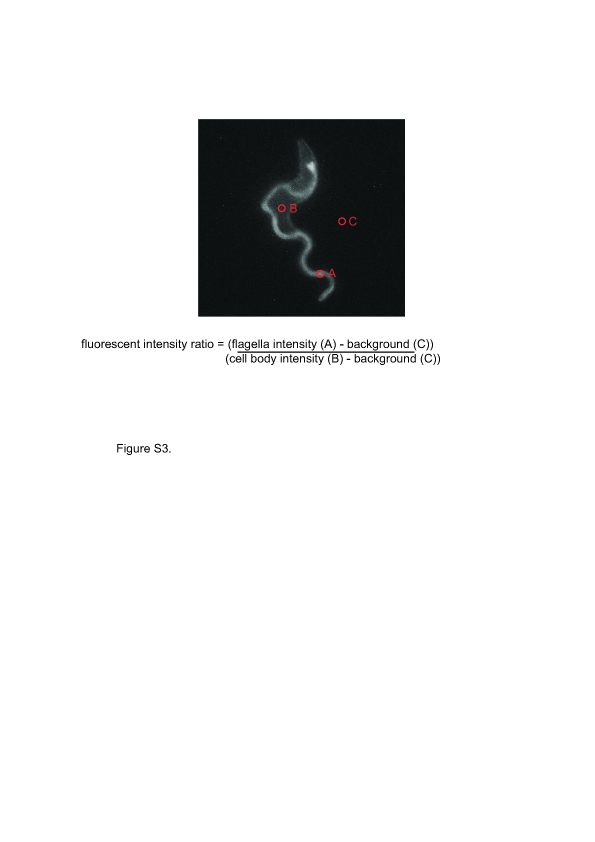

Supplement: Figure S3 — Diagram to illustrate the method to measure the fluorescent intensity ratio between the flagellum and the cell body. (TIF) [file ppat.1003566.s003.tif]

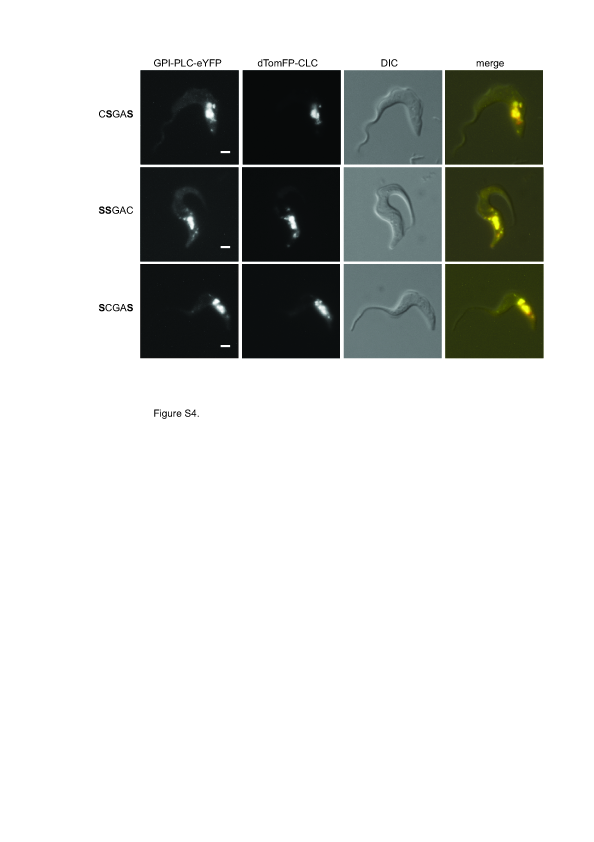

Supplement: Figure S4 — Images of representative cells expressing eYFP tagged GPI-PLC double cysteine to serine mutants with dTomatoFP tagged CLC. The CLC marks part of the endosomal system and there was clear overlap with the GPI-PLC cysteine mutants. Scale bar represents 2 µm. (TIF) [file ppat.1003566.s004.tif]

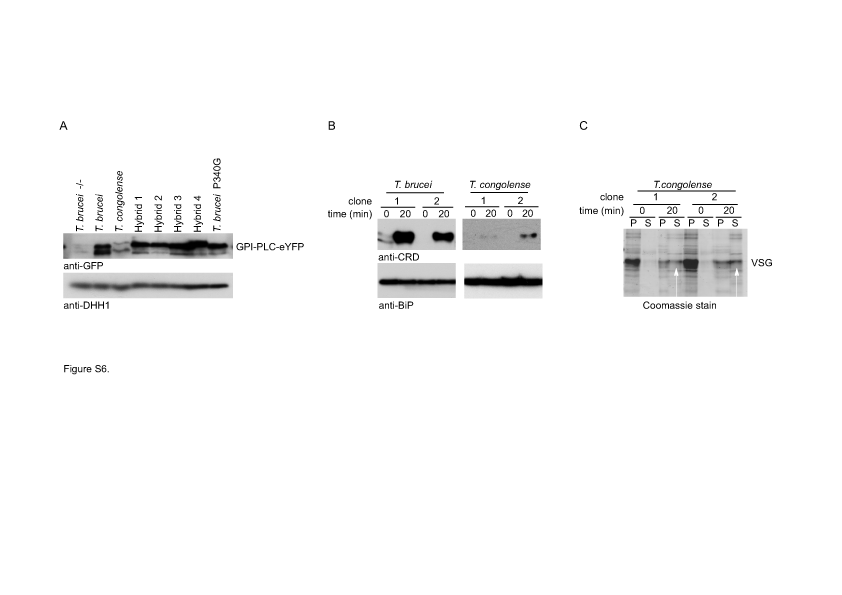

Supplement: Figure S6 — A) Western blot of cells expressing a variety of eYFP tagged GPI-PLC constructs probed with anti-GFP and anti-DHH1 (loading control). B) Western blot of detergent lysis of cells expressing T. brucei GPI-PLC and T. congolense GPI-PLC probed with anti-CRD and anti-BiP (loading control). T. congolense GPI-PLC was partially active. C) Coomassie stained gel of hypotonic lysis of cells expressing T. congolense GPI-PLC. The arrows indicated the sVSG released. P = pellet, S = supernatant. (TIF) [file ppat.1003566.s006.tif]

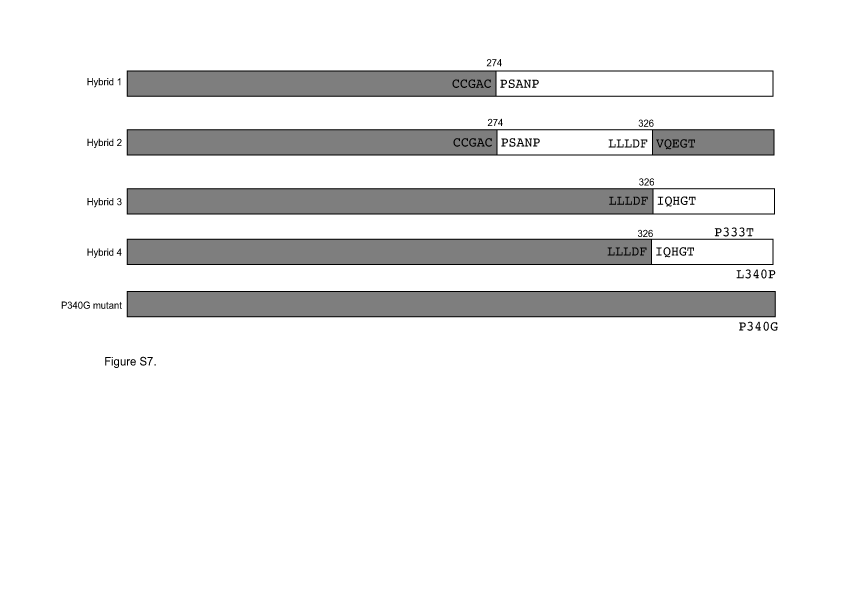

Supplement: Figure S7 — Schematic of the T. brucei and T. congolense hybrids constructed. Grey corresponds to T. brucei sequence and white corresponds to T. congolense sequence. The sequence of the hybrid at the point the sequences switch is shown within the protein and the residue number at which the switch occurs is above the sequence. Mutations are shown above and below the sequence with the original residue, its number and the residue it becomes. (TIF) [file ppat.1003566.s007.tif]

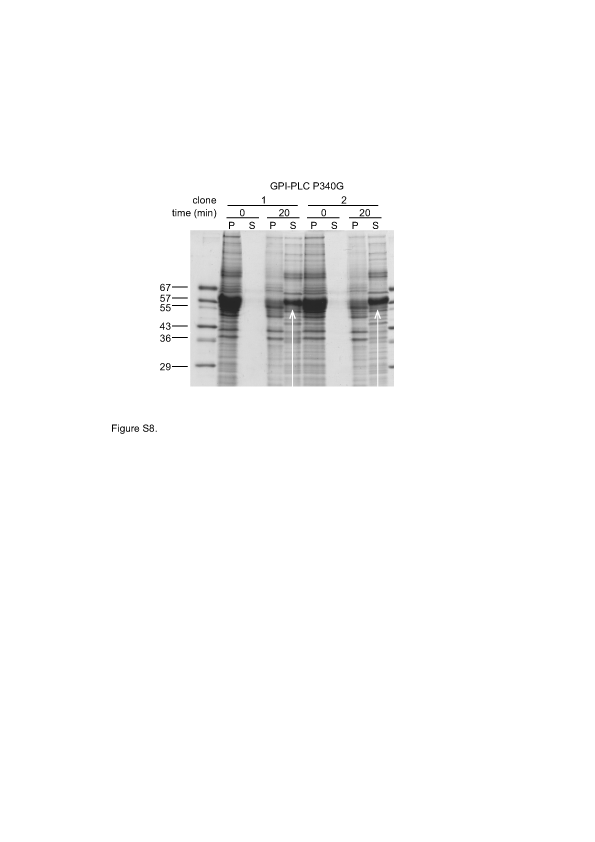

Supplement: Figure S8 — Coomassie stained gels of hypotonic lysis assays of cells expressing GPI-PLC P340G. The arrows indicated the sVSG released. P = pellet, S = supernatant. (TIF) [file ppat.1003566.s008.tif]

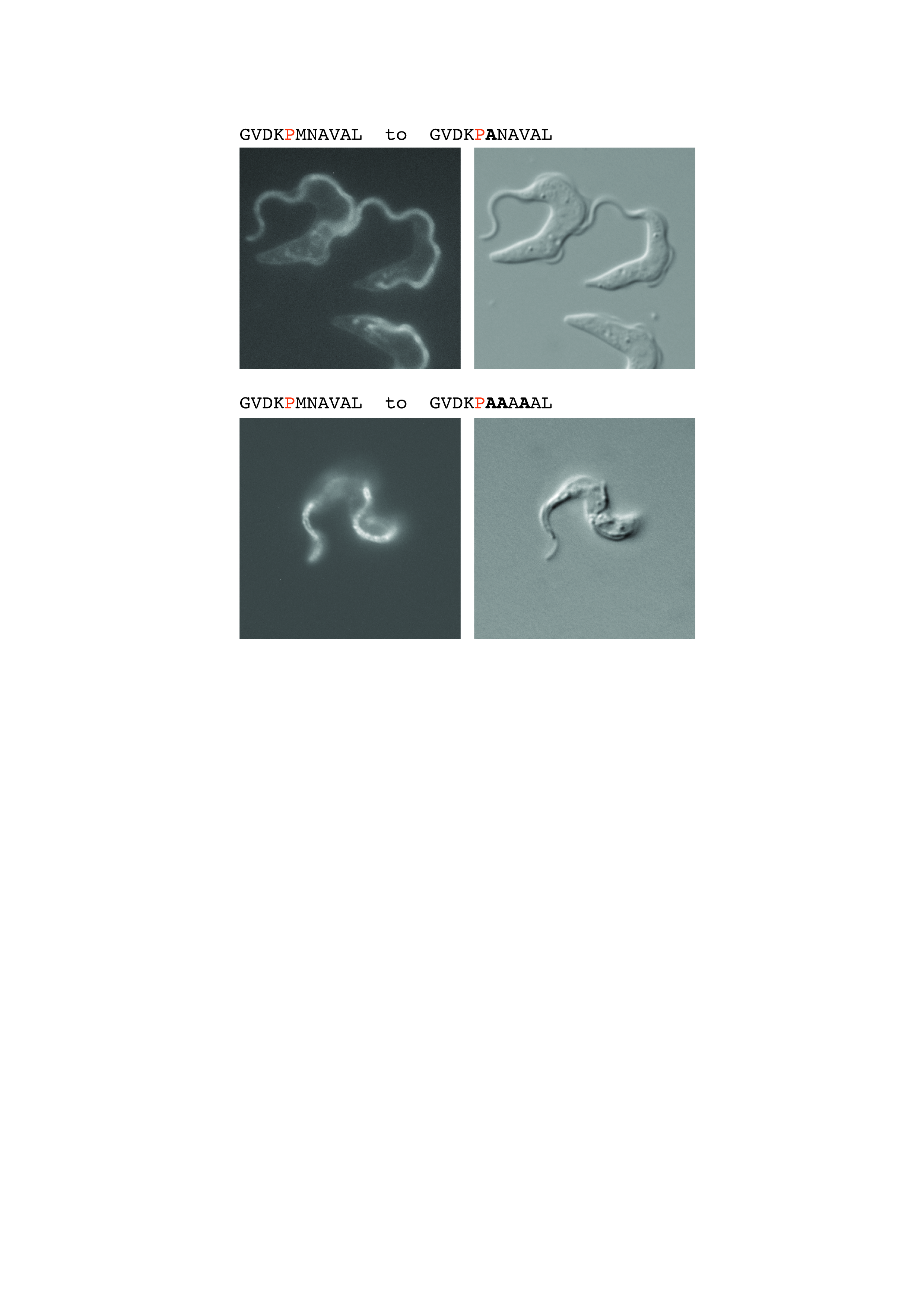

Supplement: Figure S9 — Mutations to the C-terminal side of P340 do not affect flagellar concentration of GPI-PLC. The mutations are shown above the representative images, P340 is indicated in red. (TIF) [file ppat.1003566.s009.tif]

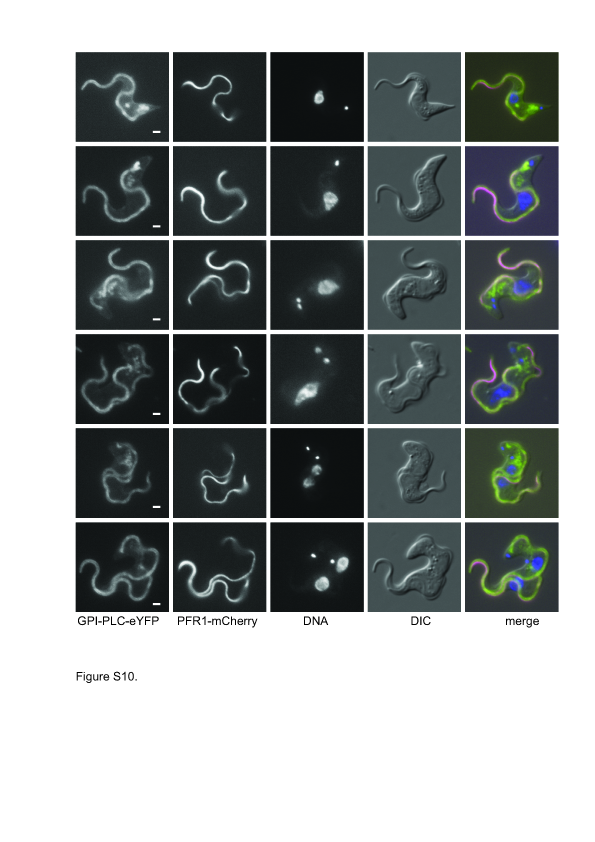

Supplement: Figure S10 — Images of typical cells expressing GPI-PLC-eYFP and PFR1-mCherryFP. 200 cells were imaged and all were similar to the cells above. Scale bar represents 2 µm. (TIF) [file ppat.1003566.s010.tif]
