# Supplementary material for: Determinants of GPI-PLC Localisation to the Flagellum and Access to GPI-Anchored Substrates in Trypanosomes
Source: PLoS Pathog. 2013 Aug 22;9(8):e1003566. doi: 10.1371/journal.ppat.1003566 (PMC3749955; doi:10.1371/journal.ppat.1003566)
Supplement: Figure S5 — Alignment of GPI-PLC from T. brucei and T. congolense. The cysteines in the CCGAC motif are shown in red. The proline residues that were mutated towards the C-terminus are also shown in red. The points at which fusion constructs were joined are highlighted in yellow. T. brucei numbering is used throughout. (DOCX) [file ppat.1003566.s005.docx]

Tbr MFGGVKWSPQSWMSDTRSSIEKKCIGQVYMVGAHNAGTHGIQMFSPFG 48

Tco MPETEGIKWSPQSWMDNLRSSIEGRAITQLFMVGAHNTGTDAIHMFSPFG

* ******** ***** * * ****** ** * ******

Tbr LDAPEKLRSLPPYVTFLLRFLTVGVSSRWGRCQNLSIRQLLDHGVRYLDL 98

Tco LDAPEAIYGMNEGVAFLLRFLTAGVSSRWARCQSMSARQLLNHGVRYLDL

***** * ******* ****** *** * **** ********

Tbr RMNISPDQENKIYTTHFHISVPLQEVLKDVKDFLTTPASANEFVILDFLH 148

Tco RLTAGANGIDRIYTTHFHASLPLKEIIVDVKNFLETPTCGNEFIILDFQH

* ******* * ** * *** ** ** *** **** *

Tbr FYGFNESHTMKRFVEELQALEEFYIPTTVSLTTPLCNLWQS--TRRIFLV 196

Tco FYGFTD-ESMGKFVEELSPLSDYLIPSNVPLTTPLSTLWRSSTTQRVFLV

**** * ***** * ** * ***** ** * * * ***

Tbr VRPYVEYPYARLRSVALKSIWVNQMELNDLLDRLEELMTRDLEDVSIGGV 246

Tco VKPTIHHPAARLRGWALRSIWLDELQLEALLERLNRLLTRELVDAPDERV

* * * **** ** *** * ** ** * ** * * *

274

Tbr PSKMYVTQAIGTPRNNDFAVAACCGACPGSHPDLYSAAKHKNPHLLKWFY 296

Tco PSKLYVTQAIGTPKNKDVAMGACCGACPSANPDLEDVAKKINSPLLSWFY

*** ********* * * * ******* *** ** * ** ***

326

Tbr DLNVNGVMRGERVTIRRGNNTHGNILLLDFVQEGTCTVKGVDKPMNAVAL 346

Tco TLNAEGTVADRPVNLKKGNNTHGNILLLDFIQHGTCPVGKNKDLVNAVGM

** * * ************* * *** * * * ***

Tbr CVHLNTNQTARS 358

Tco CVYLNTQDTARI

** *** ***

Figure S5.
