# Supplementary material for: Determinants of GPI-PLC Localisation to the Flagellum and Access to GPI-Anchored Substrates in Trypanosomes
Source: PLoS Pathog. 2013 Aug 22;9(8):e1003566. doi: 10.1371/journal.ppat.1003566 (PMC3749955; doi:10.1371/journal.ppat.1003566)
Supplement: Table S1 — Table describing the plasmids used in this study. p1906 is described in Webb et al., (2005). p2T7-177 is described in Wickstead et al., (2002). p2937 and p3121 are derivatives of p2710 and p2679 respectively (Kelly at al., 2007): p2937 contains a gene for blasticidin resitance as opposed to the gene for G418 resistance in p2710. p3121 contains an dTomatoFP tag as opposed to the mCherryFP tag in p2679. The base plasmids p3261, p3649 and p3731 were constructed for this study. p3261 is a derivative of p1906 with a BglII site introduced before the stop codon (no change in amino acid sequence) and an a GSGSGS linker followed by eYFP added to the C-terminus. p3649 is a derivative of p3261 with a SmaI site introduced downstream of the cysteine motif (no change in amino acid sequence) to allow introduction of the cysteine motif mutants. p3731 is a derivative of p3649 with a HindIII site introduced before the start codon to allow the introduction of GPI-PLC hybrids as HindIII BamHI fragments into the HindIII BglII sites. The sequences of all plasmids are available from the authors. (DOC) [file ppat.1003566.s011.doc]

| Plasmid number | Description | Base plasmid | Resistance marker |
| --- | --- | --- | --- |
| p1906 | GPI-PLC endogenous expression plasmid | n/a | G418 |
| p3225 | GPI-PLC-eYFP C-terminal endogenous tagging plasmid | p2937 | blasticidin |
| p3261 | GPI-PLC-eYFP C-terminal endogenous expression plasmid | p1906 | G418 |
| p3391 | TbFLA3 RNAi plasmid | p2T7-177 | phleomycin |
| p3393 | TbBILBO1 RNAi plasmid | p2T7-177 | phleomycin |
| p3483 | eYFP-GPI-PLC N-terminal endogenous expression plasmid | p1906 | G418 |
| p3601 | GPI-PLC-eYFP C-terminal endogenous expression plasmid with SSGAS motif | p3261 | G418 |
| p3650 | GPI-PLC-eYFP C-terminal endogenous expression plasmid with SCGAC motif | p3649 | G418 |
| p3651 | GPI-PLC-eYFP C-terminal endogenous expression plasmid with CSGAC motif | p3649 | G418 |
| p3652 | GPI-PLC-eYFP C-terminal endogenous expression plasmid with CCGAS motif | p3649 | G418 |
| p3734 | GPI-PLC expression plasmid with *T. congolense* GPI-PLC | p3731 | blasticidin |
| p3774 | GPI-PLC hydrid 1 in expression plasmid | p3731 | blasticidin |
| p3775 | GPI-PLC-eYFP C-terminal endogenous expression plasmid with CSGAS motif | p3649 | G418 |
| p3798 | GPI-PLC-eYFP C-terminal endogenous expression plasmid with SCGAS motif | p3649 | G418 |
| p3799 | GPI-PLC-eYFP C-terminal endogenous expression plasmid with SSGAC motif | p3649 | G418 |
| p3904 | GPI-PLC hybrid 3 in expression plasmid | p3731 | blasticidin |
| p3939 | GPI-PLC hybrid 2 in expression plasmid | p3731 | blasticidin |
| p4083 | GPI-PLC hybrid 4 in expression plasmid | p3731 | blasticidin |
| p4103 | TbCLC dTomato N-terminal endogenous tagging plasmid | p3121 | puromycin |
| p4110 | TbGPI-PLC ORF with P340G mutation | p3731 | blasticidin |

Table S1
